# Amygdala GluN2B-NMDAR dysfunction is critical in abnormal aggression of neurodevelopmental origin induced by St8sia2 deficiency

**DOI:** 10.1038/s41380-018-0132-3

**Published:** 2018-08-08

**Authors:** Alexandre Bacq, Simone Astori, Elias Gebara, Wei Tang, Bianca A. Silva, Jose Sanchez-Mut, Jocelyn Grosse, Isabelle Guillot de Suduiraut, Olivia Zanoletti, Catherine Maclachlan, Graham W. Knott, Johannes Gräff, Carmen Sandi

**Affiliations:** 1 Laboratory of Behavioral Genetics, Brain Mind Institute, School of Life Sciences, EPFL, Lausanne, Switzerland; 2 Laboratory of Synaptic Mechanisms, Brain Mind Institute, EPFL, Lausanne, Switzerland; 3 Laboratory of Neuroepigenetics, Brain Mind Institute, EPFL, Lausanne, Switzerland; 4 Interdisciplinary Centre for Electron Microscopy, EPFL, Lausanne, Switzerland

**Keywords:** Neuroscience, Diseases

## Abstract

Aggression is frequently observed in neurodevelopmental psychiatric disorders such as schizophrenia, autism, and bipolar disorder. Due to a lack of understanding of its underlying mechanisms, effective treatments for abnormal aggression are still missing. Recently, genetic variations in Sialyltransferase 2 (*St8sia2*) have been linked to these disorders and aggression. Here we identify abnormal aggressive behaviors and concomitant blunted fear learning in *St8sia2* knockout (−/−) mice. It is worth noting that the amygdala of *St8sia2*−/− mice shows diminished threat-induced activation, as well as alterations in synaptic structure and function, including impaired GluN2B-containing NMDA receptor-mediated synaptic transmission and plasticity. Pharmacological rescue of NMDA receptor activity in the amygdala of *St8sia2*−/− mice with the partial agonist d-cycloserine restores synaptic plasticity and normalizes behavioral aberrations. Pathological aggression and associated traits were recapitulated by specific amygdala neonatal *St8sia2* silencing. Our results establish a developmental link between *St8sia2* deficiency and a pathological aggression syndrome, specify synaptic targets for therapeutic developments, and highlight d-cycloserine as a plausible treatment.

## Introduction

Violence and aggression cause a great burden to individuals and societies, highly contributing to death, disease, disability, and socioeconomic problems worldwide [1]. Despite many ongoing efforts focusing on policies and sociocultural factors to tackle violence [1], its reduction has proven a difficult task. Treatments for aggressive individuals have repeatedly failed [2] and several forms of aggression are still considered intractable [3, 4]. Furthermore, individuals with a mental disorder—particularly schizophrenia, with an estimation of 9.9% of individuals compared with 1.6% of general population—exhibit increased risk for violence [5, 6]. Consequently, the need to better understand the neurobiological pathways to specific types of aggression has been voiced as a prerequisite for designing effective treatments [7].

Neuroimaging studies in humans are advancing the knowledge of structural, functional, and neurochemical alterations related to different forms of abnormal aggression [8–10]. However, for treatment development, there is a need for the identification of relevant molecular targets, which requires in-depth understanding of the molecular and cellular mechanisms that are at the core of explicit behavioral dysfunctions associated with abnormal aggression. Deficits in fear processing and reduced anxiety are behavioral traits considered to have a major role in the emergence of abnormal aggression in the general population and can be frequently observed in individuals with psychopathic traits [11, 12] and schizophrenia [13, 14]. Importantly, these traits can be reliably studied in rodent models.

To investigate the cellular mechanisms for key phenotypes linked to abnormal aggression, we selected *St8sia2* (also named *Siat8B*) knockout (*St8sia2*−/−) mice as a highly relevant model. Genome-wide association studies have identified variations in the *St8sia2* gene in association with several psychiatric disorders, including schizophrenia [15, 16], autism [17], and bipolar disorder [18], all of which involve social abnormalities and aggression [19]. Noticeably, a recent clinical case study has reported a severe behavioral profile comprising impaired social interactions and violence in a child with a 520 kb deletion of three genes on 15q26.1, including *ST8SIA2* [20]. Accordingly, previous studies have shown that *St8sia2*−/− mice present a schizophrenia-like phenotype [21], as well as an increased proneness to aggression [22], blunted fear learning [23], and reduced anxiety [22]. In addition, *St8sia2* gene expression in the brain can be affected by environmental experiences [24], including stress [25, 26].

ST8SIA2 (ST8 alpha-*N*-acetyl-neuraminide alpha-2,8-sialyltransferase 2) is one of the two sialyltransferases that produce polysialic acid, a sugar polymer that attaches to the neural cell adhesion molecule (NCAM) and has essential neuroplasticity roles [27, 28]. The polysialic acid–NCAM complex is highly expressed during brain development, when *ST8SIA2* expression is particularly prominent, while its expression decreases throughout the postnatal period to be minimum at adulthood [29] when it is not considered to have a major role. Accordingly, *St8sia2*−/− mice show reduced—although not abolished, due to the presence of the second sialyltransferase ST8SIA4—polysialic acid during prenatal and early postnatal periods, but normal levels (i.e., low, similar to controls) of polysialic acid throughout the brain at adulthood [22].

Based on the evidence in humans and mouse models alike, we reasoned here that *St8sia2*−/− mice might be characterized by the appearance of abnormal aggression, allowing for the investigation of underlying mechanisms. Aggression in rodents is considered abnormal when it deviates from natural rules of competition. Defining criteria for abnormal aggression in rodent models include decreased latency to attack, attacking harmless opponents such as juveniles or females, and attacks directed toward vulnerable body parts [30]. Here we found that *St8sia2*−/− mice exhibit signs of abnormal aggression that are attributable to an impairment in glutamatergic transmission and plasticity in the amygdala. In a combination of molecular, neurobiological, and pharmacological studies, we underscore amygdalar GluN2B-containing NMDA receptor (NMDAR) deficiency as a key element for the development of pathological aggression traits and demonstrate that NMDAR pharmacology effectively treats aggressive behavior and associated blunted fear memory.

## Materials and Methods

### Mice

All experiments were conducted in age-matched male *St8sia2*−/− mice and their wild-type (WT) littermates. The generation of *St8sia2* mutations has been described previously [23]. Briefly, heterozygous mice were intercrossed to obtain homozygous *St8sia2*−/− mice and WT littermates. All breeding couples had been previously backcrossed for more than 10 generations into the C57BL/6J background. Mice were housed in groups of two to five in standard plastic cages on a 12 h light/dark cycle (lights on at 07:00). Water and food were provided ad libitum. Animal care procedures were conducted in accordance with the Swiss Federal Guidelines for Animal Experimentation and were approved by the Cantonal Veterinary Office Committee for Animal Experimentation (Vaud, Switzerland).

### Brain punching

Animals were decapitated, and brain was extracted and snap-frozen in isopentane at − 45 °C and stored at − 80 °C until further processing. The brains were sectioned using a cryostat (Leica) and 100 μm-thick slices were mounted on slides. From six to eight bilateral punches of the amygdala (containing mainly the lateral (LA) and basal amygdala (BA)) were conducted using 0.5 to 1 mm punchers according to the atlas coordinates [31]. The tissue was collected in RNAase-free tubes and maintained in a − 80 °C freezer until further processing for RNA extraction and isolation.

### Trizol extraction for RNA and protein

Punches were homogenized by sonication in 100 μL of Trizol Reagent (Thermo-Fisher, Villebon sur Yvette, France) containing a protease inhibitor (Complete, Roche, Basel, Switzerland). Twenty microliters of chloroform were added and, after centrifugation, the aqueous phase (top layer) was precipitated with isopropanol and washed with ethanol 75%, to be used as RNA fraction. RNA amount was quantified using a nanodrop apparatus (Thermo-Fisher). The organic phase (bottom layer) was mixed with 3 vol/vol of acetone and centrifuged to precipitate the protein fraction. After washing with ethanol 95% supplemented with guanidine hydrocholoride 0.3 M and 2.5% glycerol, proteins were quantified using DC protein Assay (Bio-rad Laboratories AG, Cressier, Switzerland). RNA fraction was used for quantitative reverse trancription-PCR (RT-PCR) and protein fraction was processed to investigate polysialic acid levels.

### Quantitative RT-PCR

Complementary DNA was synthesized from total RNA using qScript cDNA SuperMix (Quantabio, Beverly, MA, USA) according to the supplier’s recommendations. For quantitative PCR (qPCR), reactions were performed in triplicate using SYBR Green PCR Master Mix (Applied Biosystems, Life Technologies, Warrington, Florida, USA) in an ABI Prism 7900 Sequence Detection system (Applied Biosystems, Life Technologies, Singapore). Two genes were used as internal controls: TATA-BOX binding protein (*tbp*) and eukaryotic elongation factor-1 (*eef1*). Primers for the genes of interest were designed using NCBI primer design tool. Primer sequences are listed in Supplementary Table 1. Gene expression was analyzed with the qBase 1.3.5 software using the comparative cycle threshold method.

### Polysialic acid enzyme-linked immunosorbent assay

Polysialic acid Elisa kit was purchased from Eurobio (AbCys Paris, France). In brief, 200 ng of protein were used in duplicate to assess polysialic acid levels, according to the manufacturer’s protocol.

### Behavioral analyses

For further details in all those tests, see Supplementary Materials.

#### Resident-intruder test

During the 3 days before testing, all test mice cohabitated with a naive female mouse with food and water available ad libitum. The home cages were not changed before testing. Socially experienced adult BALB/c male mice were used as intruders (one intruder per test mouse). All intruders were weighed to minimize differences in weight in each experimental group. On the test day, females were removed from the cage 30 min before testing. Then, the intruder was introduced into the cage of the test mouse for a 10 min period. All interaction sessions occurred during the dark phase of the light–dark cycle, in complete darkness, and were video-recorded with infrared cameras (Sony) for off-line scoring of behavior, using The Observer (v. 10; Noldus Technology, Wageningen, The Netherlands). The following parameters were scored: latency to attack, number of attacks, clinches, bites, and intruder position during the attacks. The time spent sniffing the intruder mouse was also scored, which provided an index of motivation for social interaction. The experimenter performing manual scoring was blind to the genotype of the experimental animals.

#### Social interaction test

For the social interaction test, WT or *St8sia2*−/− test mice were introduced with a conspecific (juvenile male or adult female C57BL/6J) to a neutral empty cage and their interaction was monitored for 30 min. The time spent sniffing was assessed, as well as the number of aggressive behaviors, such as tail rattling and attacks (bites or clinches).

#### Acoustic cue fear-conditioning test

Animals were subjected to cue conditioning, using an electric shock as the unconditioned stimulus. Training and testing took place in a fear-conditioning cage (Panlab, S.L., Barcelona, Spain) placed in a sound-attenuating chamber. The floor consisted of 20 steel rods through which an electric shock could be delivered. After 3 min of habituation to the apparatus, each animal was first exposed to a tone (30 s, 800 Hz, 80 dB) which co-terminated with a 2 s electric shock (0.5 mA). Three pairs of auditory stimulus shock were applied, separated by 1 min break. The day following the training, animals were tested for cue memory in a novel context: after 3 min of habituation, mice were exposed to three sounds (30 s, same as training) separated by 1 min. Behavior was scored from video recordings by an observer blind to genotype and treatment. The time spent freezing (no movement of the animals) was quantified to provide a measure of fear response.

### Immunohistochemistry

#### Tissue preparation

Mice were deeply anesthetized with an overdose of sodium pentobarbital (Nembutal; 40 mg/kg, intraperitoneal (i.p.)) and transcardially perfused with 40 mL of Ringer solution + Heparine, followed by 100 ml of 4% paraformaldehyde in 0.1 M phosphate buffer (pH 7.4). Brains were postfixed in the same fixative solution overnight and transferred in sucrose buffer 30% for 1 day. Thereafter, brains were frozen in cryomatrix and coronal sections (30–200 µm thick) were cut on a sliding microtome Hyrax S30 (Carl Zeiss, Oberkochen, Germany) and collected in cryoprotectant. For immunostaining, sections were pre-washed in phosphate-buffered saline (PBS), treated for 1 h with PBS + 1% bovine serum albumin (BSA) (Sigma-Aldrich, Buchs, Switzerland), 0.1% Triton X-100 (Sigma-Aldrich), and 5% normal donkey serum (NDS, Jackson ImmunoResearch Laboratories, Basel, Switzerland). Sections were subsequently incubated for 24 h at 4 °C with primary antibodies in PBS containing 0.2% BSA, 0.1% Triton X-100, and 5% NDS. After washing in PBS, sections were incubated for 2 h at room temperature with secondary antibodies in same solution as for the primary antibody. After washing, sections were mounted onto superfrost slides and coverslipped with Fluoromount (Southern Biotech, Birmingham, USA).

#### Phospho-ERK immunoreactivity

Animals were sacrificed following resident-intruder or fear conditioning (see Results section for details about timing). Primary antibodies used were rabbit anti-phospho-ERK (Cell Signaling 9101, 1/500) and mouse anti-NeuN (Millipore MAB377, 1/200). Secondary antibodies were anti-rabbit-alexa-568 and anti-mouse-alexa-647 (Abcam ab175470 and ab150107, respectively, 1/1000).

#### NMDAR subunit immunostaining

Sections were triple-labeled with rabbit anti-NeuN (Millipore ABN78, 1/500), mouse anti-NMDAR2B (Abcam ab28373, 1/500), and goat anti-NMDAR2A (abcam ab118587, 1/500). Secondary antibodies were anti-rabbit-alexa-488, anti-goat-alexa-568, and anti-mouse-alexa-647 (Abcam ab150073, ab175474, and ab150107, respectively, 1/1000).

#### Quantification

See Supplementary Materials.

### Lentivirus injection and synapse morphology

LentiONE Syn-GFP-WPRE virus was designed and produced by GEG-tech (Paris, France). Three-month-old WT and *St8sia2*−/− animals were deeply anesthetized by isoflurane inhalation (induction 4% isoflurane for 4 min and maintenance 2.5% isoflurane in O_2_ at a flow of 4 L/min) and placed in a stereotaxic apparatus (Kopf). Syn-GFP-WPRE lentivirus (0.5 μL) were infused in the amygdala. Coordinates were based on the mouse brain atlas [31], for LA–basolateral amygdala (BLA) (in mm, from bregma): Antero Posterior (A.P.) − 1, Medio Lateral (M.L.) ± 3, Dorso Ventral (D.V.) − 4.2. Head skin was closed with absorbable suture (Vicryl, 6-0, Ethicon, Johnson & Johnson, Issy les Moulineaux, France) and animals were isolated in a new cage. Three weeks after surgery, animals were anesthesized and perfused with paraformaldehyde 4%. Forty-micrometer-thick slices were cut and mounted on cover slides, with Verctashield containing 4′,6-diamidino-2-phenylindole. Images were taken using a confocal microscope (Zeiss LSM-700) and quantification was done with FIJI, as previously described [32]. Briefly, spine morphology was classified in two groups based on the maximal diameter of the spine head, as measured with Image J software: thin spines < 0.45 μm and mushroom spines > 0.45 μm. Then, the percentage of each type of dendritic spine was calculated for each examined neuron.

### Electron microscopy

Ultrastructural analysis of neuronal connectivity was assessed using serial block face scanning electron microscopy. Animals were fixed with cardiac perfusion of 2.5% glutaraldehyde and 2% paraformaldehyde in phosphate buffer and the brain vibratome sectioned in the coronal plane. Sections containing the region of interest were then heavy metal contrasted and resin embedded using the protocol previously described [33]. In brief, the sections were first stained with reduced osmium, followed by osmium alone, then thiocarbohydrazide, and a second osmium stain. They were then left overnight in uranyl acetate followed by lead aspartate before being dehydrated in alcohol and embedded in increasing concentrations of Durcapan resin. After the resin had cured, regions of interest were cut from the section and glued to a metal mounting stub, and placed inside a scanning electron microscope (Zeiss Merlin) integrated with an ultramicrotome (Gatan 3View). Approximately 300 serial images were collected at 6 nm per pixel using a beam current of 1.5 kV and dwell time of 1 μs. Images were 4000 by 4000 pixels, and 50 nm were cut after each image was acquired.

Images were aligned in the TrakEM2 software [34] in FIJI and, in the same program, synapses counted and classified according to whether they were asymmetric (glutamatergic), symmetric (presumed inhibitory), or if they were situated on a dendritic spine or directly on the dendritic shaft. The size of the synapse was estimated by drawing a circle on each synapse whose diameter matched the synapses’ maximum width. The density of synapses corresponds to the total number of synapses counted normalized by the volume (in µm^3^).

### Electrophysiological recordings

Acute coronal brain slices (250 µm-thick) containing the amygdala were prepared from mice aged between 1 week and 18 weeks. Animals were deeply anesthetized with isoflurane (if > 1 week old) and decapitated. The brain was quickly removed and cut using a vibrating tissue slicer (Campden Instruments, Loughborough, UK) in oxygenated (95% O_2_/5% CO_2_) ice-cold modified artificial cerebrospinal fluid (ACSF), containing (in mM): 105 sucrose, 65 NaCl, 25 NaHCO_3_, 2.5 KCl, 1.25 NaH_2_PO_4_, 7 MgCl_2_, 0.5 CaCl_2_, 25 glucose, 1.7 L( + )-ascorbic acid. After cutting, slices recovered for 1 h at 35 °C in standard ACSF containing (in mM): 125 NaCl, 25 NaHCO_3_, 2.5 KCl, 1.25 NaH_2_PO_4_, 1.2 MgCl_2_, 2 CaCl_2_, 18 glucose, 1.7 L( + )-ascorbic acid, and complemented with 2 sodium pyruvate and 3 myo-inositol. In the recording chamber, slices were superfused with oxygenated standard ACSF at room temperature. Pyramidal neurons identified with video-microscopy in the LA were patched in the whole-cell configuration with borosilicate glass pipettes (TW150F-3, WPI, Worcester, USA) pulled with a DMZ-Zeitz puller (Zeitz-Instruments, Martinsried, Germany). Electrical stimulation of glutamatergic afferents was delivered every 15 s with a bipolar concentric tungsten electrode (TM33CCINS, WPI) placed in the dorsal striatum medially to LA for subcortical inputs or in the external capsule for cortical inputs [35, 36]. Stimulation intensity (5–40 V, 100 µs) was adjusted to obtain monophasic responses of constant latency. For voltage–clamp recordings, pipettes (2–4 MΩ) were filled with (in mM): 120 CsGluconate, 10 CsCl, 10 HEPES, 10 phosphocreatine, 5 EGTA, 4 Mg-ATP, 0.2 Na-GTP, 2.5 QX-314-Cl^−^ (290–300 mOsm, pH 7.2–7.3). For AMPA/NMDA ratios, after recording AMPAR-mediated excitatory postsynaptic currents (AMPAR-EPSCs) at – 70 mV, the membrane potential was slowly switched to + 40 mV and DNQX (0.01 mM) was added to the perfusate to isolate the NMDA-EPSCs. Peak values of AMPA-EPSCs were divided by the amplitude of NMDA-EPSCs, measured as the mean of 3 ms around the absolute peak. For input–output curves, AMPA-EPSCs and NMDA-EPSCs were elicited as described above at increasing stimulus intensities (5–40 V with 5 V increments) in 5- to 8-week-old mice. The stimulus electrode was repositioned if no response could be detected at 15 V.

Current-clamp recordings were performed with pipettes (2–3 MΩ) filled with an intracellular solution containing (in mM): 130 K Gluconate, 10 KCl, 10 HEPES, 10 phosphocreatine, 0.2 EGTA, 4 Mg-ATP, 0.2 Na-GTP, (290–300 mOsm, pH 7.2–7.3). The extracellular perfusate was complemented with the GABA_A_R blocker picrotoxin (0.1 mM) for Long-Term Potentiation (LTP) experiments. Recordings were conducted with automatic bridge-balance of pipette resistance. Membrane potential was kept at − 65/− 70 mV using DC injections. The protocol for LTP induction consisted of four repetitions of a tetanic stimulation (1 s, 100 Hz, every 10 s) delivered, whereas the cell was depolarized to − 20 mV [36]. LTP was induced within 15 min after the establishment of the whole-cell configuration. Changes in synaptic efficacy were estimated by comparing the mean values of the initial slopes of the excitatory postsynaptic potentials (EPSPs) at 25–30 min after induction with the last 5 min of baseline recording. For both patch-clamp configurations, series resistance (*R*_s_) and input resistance (*R*_i_) were monitored throughout recordings by brief voltage or current pulses and data were rejected for changes in resistance > 20%. Membrane voltage values were not corrected for liquid junction potential. Data were acquired through a Digidata1550A digitizer. Signals were amplified through a Multiclamp700B amplifier (Molecular Devices, Sunnyvale, USA), sampled at 20 kHz and filtered at 10 kHz using Clampex10 (Molecular Devices). Clampfit10 (Molecular Devices) and Igor Pro 6 (WaveMetrics) were used for data analysis.

### Intra-cerebral cannulation surgery

Mice subjected to pharmacological experiments were implanted bilaterally with stainless steel guide cannulas aimed at the lateral ventricle (intracerebroventricularly, i.c.v.) or intra-LA. Mice were anesthetized by isoflurane (see lentivirus injection section) and placed in a stereotaxic apparatus (Kopf). Small holes were drilled through the skull for bilateral placement of stainless steel 25-gauge guide cannulae (Plastics One, Roanoke, VA, USA) fitted with a removable dummy cannula. Coordinates were based on the mouse brain atlas [31] (in mm, from bregma), for ventricle: A.P. − 0.6, M.L. ± 1.2, D.V. − 1.0; for LA: A.P. − 1, M.L ± 3, D.V. − 3.5. Cannulae were fixed to the skull with one anchoring screw and dental cement (Duralay 2244; Reliance, Worth, IL). After behavioral experiments, animals were sacrificed by i.p. pentobarbital injection and correct cannulae placement was routinely verified with Cresyl Violet histology.

### Drug infusions

Behavioral experiments were performed 5 or 20 min (see Results) after d-cycloserine (DCS, Merck) administration. We randomly assigned animals to their respective treatment, but matched the groups by anxiety (i.e., same average anxiety level between vehicle and DCS groups). For intra-cerebral infusions, the dummy was removed and an injector was inserted that extended 1 mm from the tip of the cannulae. A solution of 40 µg/µL of DCS was prepared in ACSF, as described previously [37], and infused in a total volume of 1 µL for i.c.v. and 0.3 µL for LA during 1–2 min of constant flow. The injector remained in place for one additional minute after infusion to allow proper diffusion.

### In vivo transfection of sh-St8sia2

#### Plasmids

MSH028199-CU6 short hairpin RNA (shRNA) clone set against Mouse NM_009181.2 (sh-St8sia2) and control (CSHCTR001-CU6) were purchased from genecopoeia (Rockville, USA).

#### Surgery and injection

At postnatal day 2 (P2), sh-St8sia2 or control green fluorescent protein (GFP) expression vectors were transfected directly into the amygdala by polyethylenimine (PEI)-mediated gene delivery. Plasmid DNA/PEI complexes were prepared according to the manufacturer’s protocol (in vivo-jetPEI; PolyPlus Transfection). In brief, 10 μg sh-St8sia2 or control plasmid DNA were diluted in a sterile solution of 5% glucose to a final volume of 16.8 μL and complexed with 3.2 μL of linear PEI. Pups were anesthetized with a mix of dormicum and Dormitor and placed in a stereotaxic frame (David Kopf Instruments, Tujunga, CA, USA), using a three-dimensional printed model of P2 body shape. The insertion point of the 30-gauge injection needle was 0.7 mm lateral to the superior sagittal sinus and ± 2.0 mm rostral to the lambda. The needle was inserted to a depth of 3.5 mm from the surface of the skin. PEI–plasmid complex (0.2 μL) were injected using a 5 μL Hamilton syringe. After 5 min, the needle was withdrawn slowly. The pups were injected with an anti-sedative, placed on a heating pad with their original nesting material for 3–5 min, and then returned to their mother for further recovery. The accuracy of the coordinates was determined in pilot experiments with infusion of methylene blue dye.

### Prenatal stress Paradigm

Pregnant mothers (C57BL/6J) were placed into a well-ventilated 50 mL centrifugal tube under bright light for 45 min per day during the last 6 days of pregnancy. E18 male embryos were collected immediately after the last restraint stress session and brains frozen. Control mothers were left undisturbed in home cages.

## Results

### Abnormal aggression in St8sia2−/− mice is associated with impaired amygdala activation

First, we verified that *St8sia2* expression and polysialic acid levels throughout the brain in WT mice are high during the neonatal period (postnatal day 5, P5) and decay afterwards (as detected from P15 onwards; Supplementary Figure 1a-b). These findings are consistent with previous evidence [29]. During the neonatal period (P4), we confirmed that *St8sia2* expression levels are high in WT mice, while absent in *St8sia2*−/− mice, and *St8sia4* mRNA levels not significantly different between the two genotypes (Supplementary Figure 1c). Therefore, in experiments reported below in which mice are tested at adulthood, *St8sia2*−/− mice present *St8sia2* and polysialic acid levels equivalent to controls, and any detected change in their behavior or brain features is likely to be of developmental origin.

**Fig. 1:**
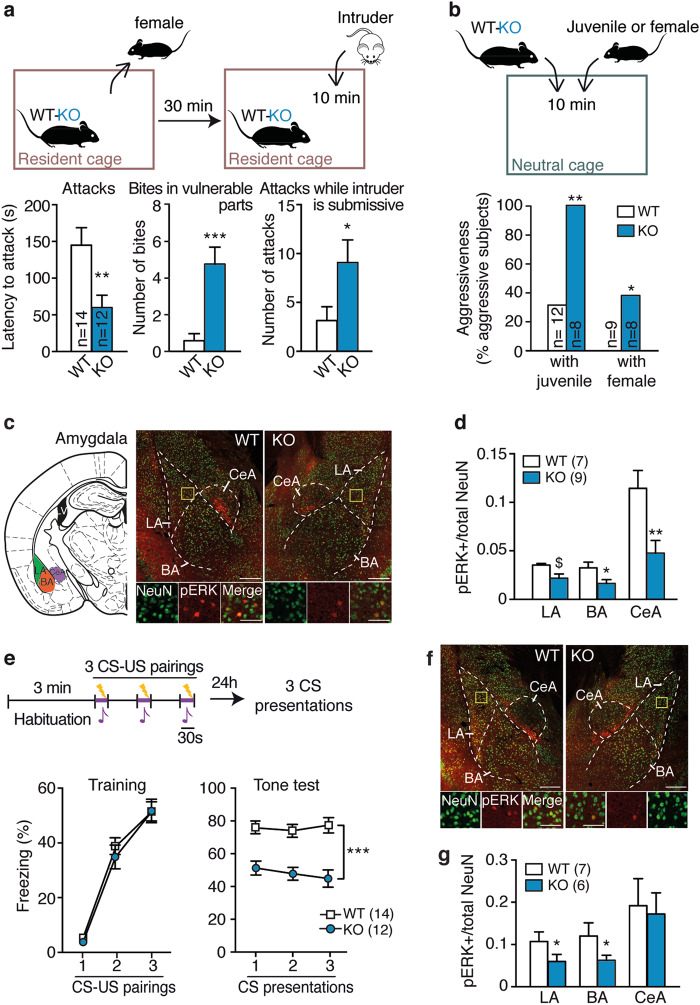
Abnormal aggression in *St8sia2*−/− mice is associated with impaired amygdala activation. **a** Abnormal aggressive behaviors in *St8sia2*−/− (KO), as compared with wild-type (WT) mice, in the resident-intruder test (protocol at the top) as shown in latency to attack (left, unpaired *t*-test, *t*_24_ = 2.81, *p* = 0.0096), number of bites in vulnerable body parts (middle, Mann–Whitney test, *U* = 10.5, *p* < 0.0001) and number of attacks, while intruders are in submissive postures (right, Mann–Whitney test, *U* = 42.5, *p* = 0.024). **b** Abnormal aggression of *St8sia2*−/− mice against juvenile (*χ*^2^-test = 8.89, *p* = 0.0029) and female (*χ*^2^-test = 4.098, *p* = 0.043) conspecifics, assessed with a social interaction test (protocol at the top). **c** Representative immunofluorescence for phospho-ERK (pERK) activation (red) in neurons marked by NeuN (green) from mice submitted to a resident-intruder test and localization for quantification in amygdala (top; LA lateral, BA basal, CeA central). **d** Quantification of pERK activation after resident-intruder test in amygdala (unpaired *t*-test, *t*_14_ = 1.97, *p* = 0.069 for LA; *t*_14_ = 2.39, *p* = 0.03 for BA; *t*_14_ = 3.027, *p* = 0.0097 for CeA). **e** Acoustic fear conditioning in *St8sia2*−/− (KO) and WT mice [protocol at the top; CS conditioned stimulus (tone); US unconditioned stimulus (foot-shock)], including the training phase (left, two-way ANOVA: main effect of CS-US, F_2,48_ = 111.3, *p* < 0.0001) and the tone test (right, two-way ANOVA: main effect of genotype, F_1,24_ = 106.6, *p* < 0.0001). **f** Representative immunofluorescence for pERK activation (red) in neurons (green) from mice submitted to fear conditioning. **g** Quantification of pERK levels after fear conditioning in *St8sia2*−/− (KO) and WT mice in amygdala (LA: unpaired *t*-test, *t*_11_ = 1.18, *p* = 0.05; BA: *t*_1__1_ = 2.33, *p* = 0.04). Scale bars, 100 µm, insert: 20 µm. Results are given as mean ± SEM. ^$^*p* = 0.06, *p < 0.05; **p < 0.01; ***p < 0.001 vs. WT

When compared with their WT littermates during social encounters (Fig. 1a; see Supplementary Figure 2 for experimental timeline), *St8sia2*−/− mice displayed higher signs of abnormal aggression, according to the defining criteria for abnormal aggression in rodents [30]. These included higher aggression levels toward adult male intruders in their homecage, shorter attack latencies than WT mice, and higher occurrence of biting in vulnerable body parts, as well as attacks, while the intruder is displaying submissive postures (Fig. 1a, Supplementary Movie 1). In addition, when placed in a novel environment along with either a naïve juvenile or a female conspecific —two experimental conditions that do not pose a real threat and typically induce little or no aggressiveness—*St8sia2*−/− mice showed significantly higher aggressiveness than WT mice (Fig. 1b). Social investigation did not differ between genotypes in both tests, as estimated from sniffing behavior (Supplementary Figure 3a-b). Altogether, these observations point at a pathological aggression phenotype [30] in *St8sia2*−/− mice, not previously characterized.

We next examined behavior-induced activation of several brain regions implicated in pathological aggression, such as the amygdala, the prefrontal cortex, and the ventromedial hypothalamus (VMH), as alterations in these brain regions have been linked with pathological aggression [38, 39] and psychopathy in humans [40–42]. To this end, we measured a dynamic marker of neuronal activation, the phosphorylation of extracellular signal-regulated kinases 1 and 2 (pERK) [43], which was shown to be critically expressed in social brain circuits by aggressive interactions [44]. Following a resident-intruder test, *St8sia2*−/− mice exhibited lower pERK levels in the LA, BA, central amygdala (Fig. 1c, d), and medial amygdala (Supplementary Figure 3c) nuclei, as compared with their WT littermates (see Supplementary Figure 3d-e for behavioral data). However, no significant genotype-related differences were evident in any of the prefrontal cortex subdivisions [infralimbic, prelimbic or cingulate] (Supplementary Figure 3f), or in the VMH (Supplementary Figure 3g). Importantly, corticosterone levels following the resident-intruder test were comparable between genotypes (Supplementary Figure 3h), suggesting that the observed pERK changes were not due to differences in stress responses.

When tested for fear processing, *St8sia2*−/− mice showed a deficit in making acoustic fear memories. During fear conditioning, both genotypes reacted with comparable increments in freezing behavior (Fig. 1e). However, when presented with the acoustic stimulus in a new context 24 h afterwards, *St8sia2*−/− mice displayed a significantly reduced freezing time as compared to WT mice, indicating decreased fear memory (Fig. 1e). This alteration was not due to deficits in the auditory capacities or in pain sensitivity, as *St8sia2*−/− mice showed equivalent startle responses to WT levels when exposed to different acoustic stimuli (Supplementary Figure 3i) and comparable reaction to foot shocks (Supplementary Figure 3j). Given the essential roles played by the amygdala and prefrontal cortex in acoustic fear memories [45], we also assessed activation of these areas following fear conditioning. *St8sia2*−/− mice showed reduced pERK levels throughout the LA and BA nuclei of amygdala than WT mice (Fig. 1f, g, see Supplementary Figure 3k for behavioral data), while not differing in the prefrontal cortex (Supplementary Figure 3l), or in the auditory cortex (Supplementary Figure 3m).

Then, we obtained strong evidence that *St8si2a*−/− mice display a hypoanxious phenotype across different tests, as they spent more time in the anxiogenic open arm of the elevated plus maze (Supplementary Figure 3n), more time in the anxiogenic center of the open field (Supplementary Figure 3o) and buried less marbles in the marble test (Supplementary Figure 3p). Locomotor activity, measured during 24 h in activity cage, was not different between genotypes (Supplementary Figure 3q).

Therefore, *St8sia2*−/− mice present a phenotype characterized by abnormal aggression, alongside deficits in fear processing, reduced anxiety, and impaired amygdala activation, which are reminiscent of symptoms characterizing psychopathic subjects [46, 47] and violent schizophrenic patients [48].

### Neurobiological alterations in the LA induced by St8sia2 deficiency

**Fig. 2:**
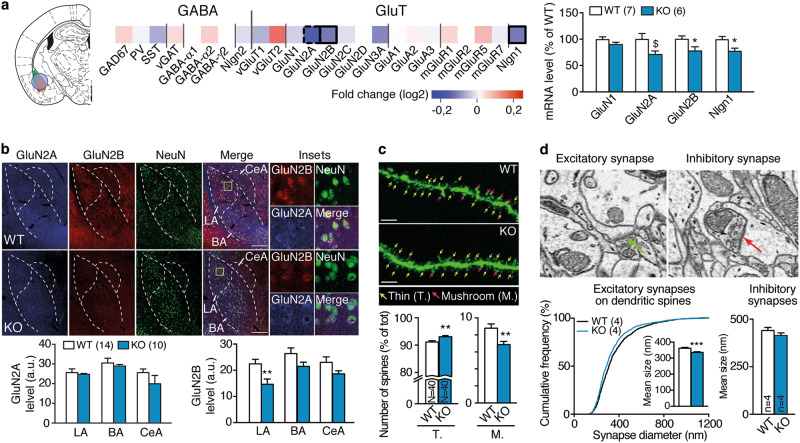
Neurobiological alterations in the lateral amygdala induced by *St8sia2* deficiency. **a** Heatmap representation of GABAergic and glutamatergic (GluT) gene markers in the amygdala of *St8sia2*−/− (KO) mice relative to wild-type (WT) levels (bordered squares represent significant differences, the doted one represent a trend; labeling refers to the protein expressed by the related gene). On the left, the location of the punch is represented. Quantification of mRNA levels of genes with altered expression is provided on the right panel (unpaired *t*-tests, *t*_11_ = 2.064, *p* = 0.063 for GluN2A; *t*_11_ = 2.28, *p* = 0.043 for GluN2B; *t*_11_ = 2.45, *p* = 0.037 for Nlgn1). **b** Representative pictures of immunostaining (top, showing GluN2A (blue), GluN2B (red), and NeuN (green)), and corresponding quantification (bottom) in amygdala of *St8sia2*−/− and WT mice (LA lateral, BA basal, CeA central amygdala) (unpaired *t*-test, *t*_22_ = 2.932, *p* = 0.0077 for LA). Scale bars, 100 µm. **c** Representative images of GFP-labeled neurons of WT (top) and *St8sia2*−/− (bottom), with thin (T.) and mushroom-like (M.) spines identified by color-coded arrows, and quantification (*n* = 4 mice per genotype, *N* = 40 segments of neuron per genotype, unpaired *t*-test, *t*_78_ = 3.054, *p* = 0.0031 for thin; *t*_78_ = 3.054, *p* = 0.0031 for mushroom-like spines). **d** Representative image of electron microscopy of an excitatory/asymmetric spine on the left and a symmetric/inhibitory synapse on the right (arrows point the synaptic cleft). Graphs, quantification of the distribution of the size of excitatory synapses (left, Kolmogorov–Smirnov test on cumulative distributions, *D* = 0.089, *p* < 0.001, and Mann–Whitney test on mean size values, *U* = 815239, *p* < 0.0001, *n* = 4 mice per genotype, *N* = 1229–1484 synapses) and of the size of inhibitory synapses (right, Mann–Whitney test, *U* = 17376, *p* = 0.11, *n* = 4 mice per genotype, *N* = 191-201 synapses) in lateral amygdala of *St8sia2*−/− and wild-type (WT) mice. Results are given as mean ± SEM. ^$^*p* = 0.06, *p < 0.05; **p < 0.01; ***p < 0.001 vs. WT

Our data point to a deficit in amygdala function in *St8sia2*−/− mice. We next investigated whether these mice present alterations at the synaptic level in the amygdala. First, we examined expression levels of glutamatergic- and GABAergic-related genes by RT-qPCR in amygdala punches (containing mainly the LA and BA, see Fig. 2a) and found evidence for alterations in glutamatergic ones. Specifically, *St8sia2*−/− mice had lower mRNA levels of the NMDAR subunits GluN2A and GluN2B than WT mice, as well as Neuroligin1—a cell adhesion molecule associated to glutamatergic synapses—(Fig. 2a). Quantification of protein content by immunohistochemistry confirmed the GluN2B, but not GluN2A, reduction in *St8sia2*−/− mice, and particularly in the LA (Fig. 2b). Noteworthy, we did not find any differences in the density of GABAergic and glutamatergic neurons (Supplementary Figure 4a). To assess whether these observations were paralleled by structural differences, we then investigated the morphology of LA pyramidal neurons using GFP-labeling induced by lentivirus injection (Supplementary Figure 4b). *St8sia2*−/− mice presented alterations in dendritic spine morphology, showing more thin and less mushroom-like spines than WT mice (Fig. 2c). To assess whether this difference corresponds to alterations in synapse morphology, we used serial section electron microscopy to investigate the size and number of excitatory synapses on dendritic spines. Although synapse density did not change (Supplementary Figure 4b-c), excitatory synapses were smaller in *St8sia2*−/− mice and inhibitory synapses did not differ (Fig. 2d). Thus, *St8sia2*−/− mice display reduced expression of GluN2B along with structural signs of weakened glutamatergic synapses in the LA.

### NMDAR dysfunction in LA of St8sia2−/− mice

**Fig. 3:**
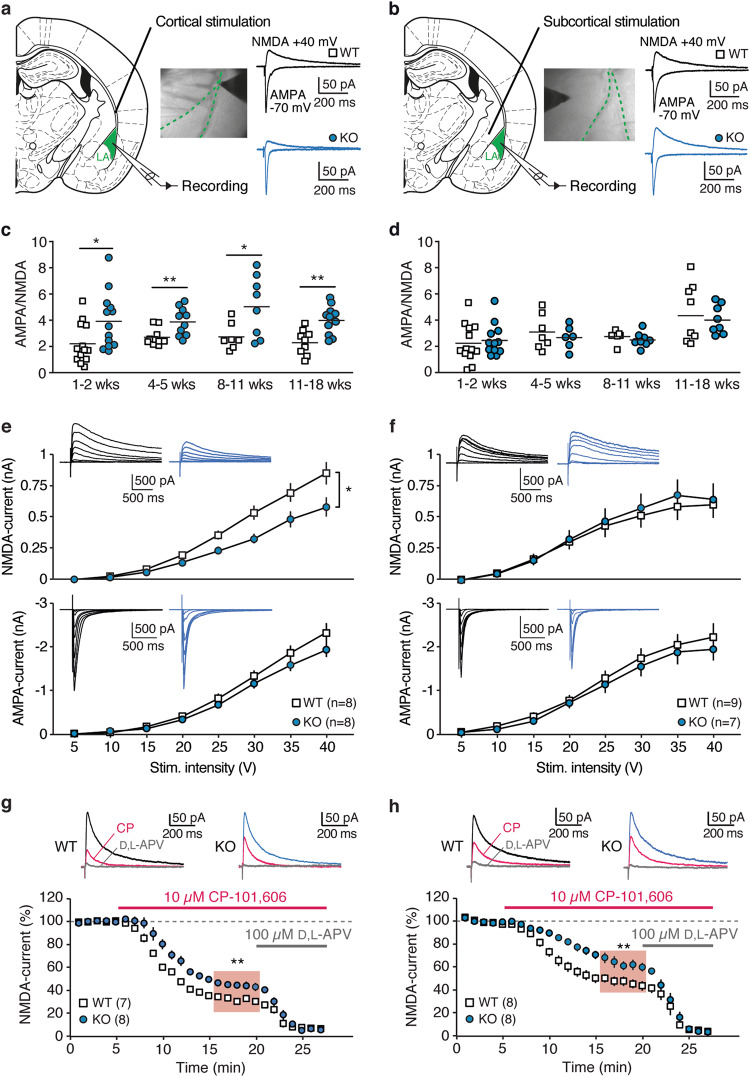
NMDAR dysfunction in lateral amygdala of *St8sia2*−/− mice. **a**–**f** AMPA- and NMDA currents were recorded in pyramidal neurons in the lateral amygdala (LA) at cortical (left column) and subcortical (right column) inputs. **a**, **b** Wide-field images and diagrams indicate typical positioning of the bipolar electrode for stimulation of cortical (**a**) and subcortical (**b**) afferents. Representative synaptic currents evoked at − 70 mV (AMPA) and + 40 mV after AMPAR blockade (NMDA) are shown for wild-type (WT, black) and *St8sia2*−/− (KO, blue) mice. **c** At cortical inputs, AMPA/NMDA ratio was measured during early postnatal stage (1–2 weeks: unpaired *t*-test, *t*_24_ = 2.31, *p* = 0.029), juvenile period (4–5 weeks: Mann–Whitney test, *U* = 18, *p* = 0.0076), young adults (8–11 weeks, *t*_13_ = 2.41, *p* = 0.031) and in older mice (11–18 weeks: *t*_18_ = 3.67, *p* = 0.0017). **d** Same measurements as in **c** for subcortical inputs (*p* > 0.05 at all developmental stages). **e**, **f** Input–output curves of NMDA currents (top) and AMPAR currents (bottom) elicited at cortical (**e**) and subcortical (**f**) inputs with increasing stimulus intensities, revealing decreased NMDAR-mediated output at cortical synapses from *St8sia2*−/− mice (two-way ANOVA: main effect of genotype: F_1,14_ = 5.97, *p* = 0.0284). Color-coded traces are representative mean currents (average of four traces) elicited at the different intensities. **g**, **h** Effect of GluN2B-specific antagonist CP-101,606 (10 µM) on cortically (**g**) and subcortically (**h**) evoked NMDAR currents in WT and *St8sia2*−/− mice (unpaired *t*-test for mean values in the shaded area, *t*_13_ = 3.28, *p* = 0.006 for **g**, and *t*_14_ = 3.006, *p* = 0.0094 for **h**). Traces at the top are NMDAR currents during baseline, after superfusion of CP-101,606 (magenta) and of the NMDAR blocker D,L-APV (100 µM), which abolished the residual current (gray). Results are given as mean ± SEM. *p < 0.05; **p < 0.01

Following the observed structural changes, we measured functional synaptic plasticity using patch-clamp recordings in acute brain slices. We first verified that LA pyramidal neurons from *St8sia2*−/− mice had comparable intrinsic excitability to WT mice (Supplementary Figure 5a-b). We also did not find differences in miniature inhibitory currents (Supplementary Figure 5c), consistent with the results from gene expression and structural analyses that indicated no altered GABAergic inputs. We then examined synaptic responses evoked at cortical inputs, through stimulation of the external capsule, and at subcortical inputs, mainly of thalamic origin, through stimulation in the dorsal striatum medially to LA (Fig. 3a, b) [49]. Values of AMPA/NMDA ratio were increased at cortical inputs of *St8sia2*−/− mice from the first postnatal week until adulthood (Fig. 3a, c). By contrast, AMPA/NMDA ratios at subcortical afferents were comparable between genotypes at all developmental stages (Fig. 3b, d). Synaptic alterations were not accompanied by changes in paired-pulse ratio of currents elicited by pairs of stimuli (50 ms inter-stimulus interval), indicating preserved presynaptic function in *St8sia2*−/− mice (Supplementary Figure 5d-e). Changes in AMPA/NMDA ratio at cortical inputs were most likely due to a decrease in NMDAR-mediated currents in *St8sia2*−/− mice. First, input–output curves of glutamatergic currents elicited by increasing stimulus intensities revealed a significant reduction in NMDAR-mediated output at cortical, but not at subcortical synapses, whereas AMPAR-mediated currents were comparable at either inputs for both genotypes (Fig. 3e, f). In addition, we verified that quantal AMPAR currents induced by asynchronous release at cortical inputs in the presence of Sr^2+^ had comparable peak amplitudes (Supplementary Figure 5f). Notably, NMDAR currents in *St8sia2*−/− mice displayed lower sensitivity to the GluN2B-selective blocker CP-101,606 (10 µM), regardless of the stimulated input (Fig. 3g, h). This indicates a reduced contribution of GluN2B to the synaptic population of NMDARs and is consistent with the observed downregulation in the corresponding gene and protein levels (Fig. 2a, b).

**Fig. 4:**
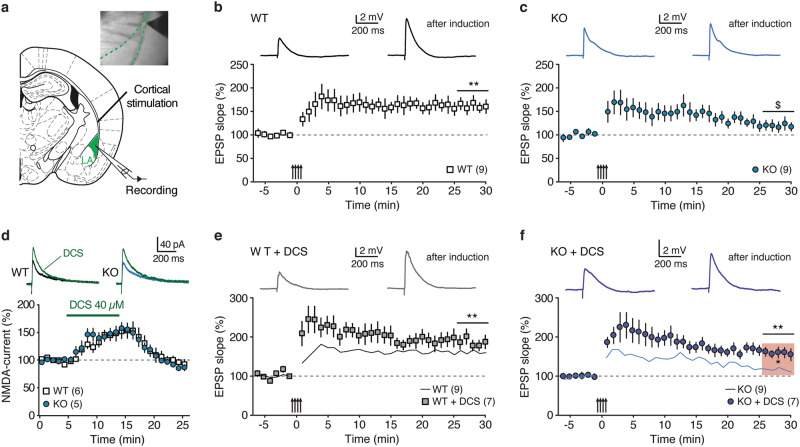
Impaired synaptic plasticity in lateral amygdala of *St8sia2*−/− mice is normalized by d-cycloserine (DCS). **a** Experimental scheme for LTP induction at LA cortical synapses in acute slices. **b**, **c** Tetanic stimulation paired with postsynaptic depolarizations induced robust Hebbian LTP at cortical inputs in wild-type (WT) mice, but failed to elicit persistent potentiation in *St8sia2*−/− (KO) mice. Arrows indicate tetanic stimulation. Top, mean excitatory postsynaptic potentials (EPSPs) during baseline and after induction. Significant level of LTP was assessed by comparing absolute EPSP slope values in the time window indicated by the horizontal bars with baseline values. **d** Effect of acute application of DCS (40 µM) on NMDAR currents in WT and *St8sia2*−/− mice (Mann–Whitney test between genotypes, *U* = 14, *p* = 0.9). Representative NMDAR currents are shown at the top (color-coded). **e**, **f** Effect of DCS on LTP induction in WT and *St8sia2*−/− mice. Solid lines reproduce data series from **b** and **c**, for comparison. Notably, DCS restored sustained potentiation in KO animals (Mann–Whitney test KO vs. KO + DCS, *U* = 11, *p* < 0.05, indicated by the shaded area in **f**). Results are given as mean ± SEM. ^$^*p* < 0.1; *p < 0.05; **p < 0.01

NMDARs —particularly GluN2B-containing— have a key role in the induction of LTP in the BLA, a requirement for the establishment of auditory fear memories [35, 50–52]. To test the hypothesis of dysfunctional synaptic plasticity in the LA of *St8sia2*−/− mice, we examined Hebbian synaptic plasticity at cortical afferents, as this input exhibited the most prominent deficits in glutamatergic transmission (Fig. 4a). In WT mice, pairing tetanic stimulations with postsynaptic depolarization [36] resulted in robust LTP of EPSPs (EPSP initial slope change 25–30 min after induction: 61 ± 12%, *n* = 9, *p* < 0.01; Fig. 4b), which was prevented by NMDAR blockade with 100 µM D,L-2-Amino-5-phosphonopentanoic acid (APV) (8 ± 6%, *n* = 6, *p* > 0.05; Supplementary Figure 5g). Strikingly, tetanic stimulation was less effective in *St8sia2*−/− mice (EPSP initial slope change: 19 ± 10%, *n* = 9, *p* = 0.056; Fig. 4c) and the level of potentiation was significantly reduced when compared with WT mice (*p* < 0.05; see Supplementary Figure 5h for comparison between series). Given the mechanistic involvement of LTP-like mechanisms in the LA/BA in fear memory formation [53–56], the identified deficit in GluN2B signaling and associated plasticity in *St8sia2*−/− mice seems to be at the core of their fear-conditioning deficits.

### DCS treatment normalizes synaptic dysfunctions and abnormal aggression

DCS is a partial agonist of NMDARs used in the clinic to aid behavioral psychotherapies to treat anxiety disorders in humans [57, 58]. To assess whether DCS could ameliorate the synaptic plasticity deficits identified in *St8sia2*−/− mice, we first performed experiments in acute slices (Fig. 4d–f). In both WT and *St8sia2*−/− mice, DCS (40 µM) reversibly increased NMDAR currents in a comparable fashion (Fig. 4d). Notably, DCS was efficient in reestablishing sustained LTP in *St8sia2*−/− mice (EPSP initial slope change 59 ± 12%, *n* = 7, *p* < 0.01; Fig. 4f and Supplementary Figure 5h for comparison between series). By contrast, the level of potentiation in WT mice was only slightly augmented, suggesting the LTP might be close to saturation in control animals (84 ± 16%, *n* = 7, *p* < 0.01; Fig. 4e).

**Fig. 5:**
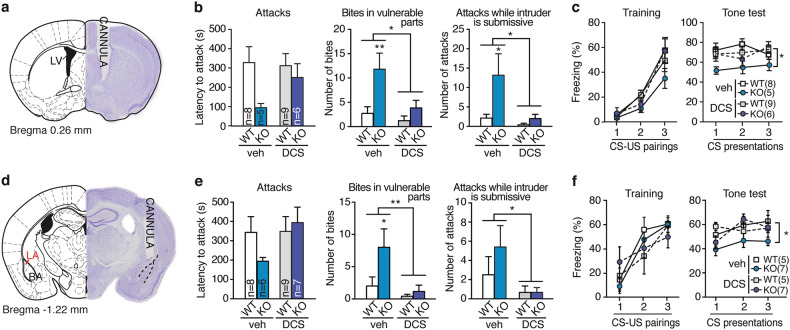
d-cycloserine treatment normalizes abnormal aggression and blunted fear memory. **a**–**c** Effect of acute i.c.v. DCS infusion (cannula implanted in the lateral ventricle (LV), **a**) on abnormal aggressiveness (**b**, two-way ANOVA: treatment effect for vulnerable bites: F_1,24_ = 6.14, *p* = 0.023 and for submissive attacks: F_1,24_ = 6.46, *p* = 0.019, Bonferroni post hoc tests) and acoustic fear conditioning (**c**, right, MANOVA: interaction factor “genotype × treatment”: F_1,24_ = 4.69, *p* = 0.041) in wild-type (WT) and *St8sia2*−/− (KO) mice. **d**–**f** Effects of acute DCS intra-amygdala infusion (cannula implanted in the lateral amygdala, **d**) on abnormal aggressiveness (**e**, two-way ANOVA: interaction factor “treatment × genotype” for bites in vulnerable parts: F_1,26_ = 5.071, *p* = 0.039; treatment effect for submissive attacks: F_1,26_ = 5.41, *p* = 0.035, Bonferroni post hoc tests) and acoustic fear conditioning (**f**, right, MANOVA: interaction factor “genotype x treatment”: F_1,20_ = 3.59, *p* = 0.02) Results are given as mean ± SEM. *p < 0.05; **p < 0.01

Based on the successful rescue of LA plasticity with DCS, we hypothesized that pharmacological facilitation of NMDAR function by treating *St8sia2*−/− mice with DCS in vivo could also normalize the pathological aggression phenotype and the blunted fear memory. To test this hypothesis, mice were cannulated and tested for behavior 2 weeks after surgery (Supplementary Figure 6a). We first applied the drug i.c.v. 20 min before behavioral testing (Fig. 5a, Supplementary Figure 6a). In *St8sia2*−/− mice, DCS normalized abnormal aggressive behaviors in the resident-intruder test (Fig. 5b) and acoustic fear memory formation (Fig. 5c). The same treatment had little or no effect in WT mice. Then, we asked whether DCS would be effective when specifically administered to the LA (bilateral infusions, 10 µg per side, Fig. 5d). Notably, all signs of pathological aggressive behavior typically observed in *St8sia2*−/− mice (i.e., latency to attack, bites in vulnerable body parts and attacks, while the intruder is submissive) were normalized by DCS treatment (Fig. 5e). Acoustic fear memory was also rescued by intra-amygdala DCS infusion (Fig. 5f) given 5 min prior to conditioning, whereas administration 20 min prior to testing was not effective (Supplementary Figure 6b). To verify the proplastic action of local DCS, pERK activation was measured *ex vivo* in brains from mice injected with DCS or vehicle before sacrifice. As expected, DCS induced significant activation of LA neurons as compared with vehicle regardless of genotype (Supplementary Figure 6c-d).

We finally tested the effect of DCS on anxiety-related behavior. Of note, animals used for DCS experiments were first tested in the open field and then assigned to drug or vehicle treatment in order to obtain groups with balanced anxiety levels (Supplementary Figure 6e, h). DCS tended to decrease anxiety in the elevated plus maze regardless of genotype for intra-amygdala infusion (Supplementary Figure 6f), whereas i.c.v. infusion of DCS effectively normalized *St8sia2*−/− mouse anxiety levels (Supplementary Figure 6i). By contrast, social preference [22] was not affected by DCS treatment (Supplementary Figure 6g, j). Therefore, DCS treatment in *St8sia2*−/− mice ameliorated their impaired amygdalar LTP and normalized their behavioral dysfunctions indicative of pathological aggression.

### Neonatal St8sia2 downregulation in the amygdala leads to abnormal aggression and associated behavioral traits

**Fig. 6:**
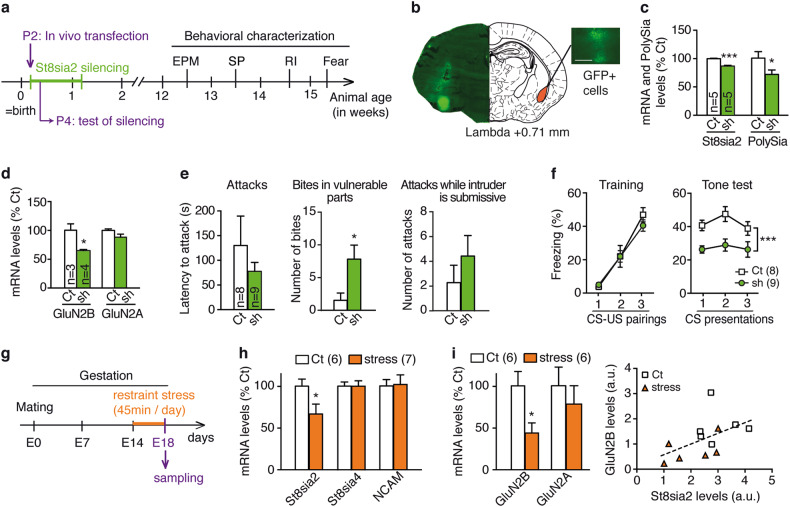
Perinatal alterations in *St8sia2*: amygdala-specific *St8sia2* knock down causes abnormal aggression, while stress induces a decrease in *St8sia2* expression. **a** Protocol for in vivo transfection at postnatal day 2 (P2) to induce temporal and spatial restricted *St8sia2* silencing and behavioral characterization (EPM elevated plus maze, SP social preference, RI resident-intruder). **b** GFP-positive cells are visible 2 days after local injection in the amygdala of control (Ct) and *St8sia2*-silencing shRNA plasmids (sh). Scale bar, 100 μm. **c** Quantification of mRNA levels of *St8sia2* and Polysialic acid (PolySia) at P4 in the amygdala of Ct and sh animals (unpaired *t*-test: *t*_8_ = 8.24, *p* = 0.0004 for mRNA; *t*_8_ = 2.15, *p* = 0.05 for PolySia). **d** mRNA quantification of GluN2B and GluN2A at P4 in the amygdala (unpaired *t*-test: *t*_5_ = 3.622, *p* = 0.0152 for GluN2B). **e**, **f** Behavioral characterization of adult animals silenced during early development. **e** Abnormal aggressive behaviors assessed by resident-intruder test in Ct and sh animals (middle, Mann–Whitney test, *U* = 6, *p* = 0.016). **f** Fear conditioning in Ct and sh animals, including training (left, two-way ANOVA: main effect of tone, F_2,30_ = 56.42, *p* < 0.0001) and tone test (right, two-way ANOVA: main effect of genotype, F_1,15_ = 19.97, *p* = 0.0005). **g** Prenatal stress (PNS) protocol. **h** Effect of PNS on *St8sia2*, *St8sia4* and NCAM mRNA expression (unpaired *t*-test, *t*_11_ = 2.23, *p* = 0.04 for *St8sia2*) at embryonic day 18 (E18). **i** GluN2B and GluN2A mRNA levels measured in control (Ct) and stress group (left, unpaired *t*-test, *t*_10_ = 2.69, *p* = 0.023) and correlation between *St8sia2* levels and GluN2B expression (linear regression, *p* = 0.071). (Control group: mothers *N* = 2, embryos *n* = 6; stress group: mothers *N* = 2, embryos *n* = 7). Results are given as mean ± SEM. *p < 0.05; ***p < 0.001 vs. control

Our data so far identifies GluN2B-related dysfunctional plasticity as a key target to rescue abnormal aggression and associated traits observed in *St8sia2*−/− mice. Here, given our findings above pointing out at the critical involvement of the amygdala in the abnormal aggression phenotype, we asked whether developmental *St8sia2* deficiency specifically in the amygdala is sufficient for the emergence of a pathological aggression phenotype. To this end, we performed local *St8sia2* gene silencing in the amygdala during the early postnatal period (Fig. 6a). At P2, we transfected a plasmid containing a shRNA designed to silence *St8sia2* in the amygdala of C57BL/6 J mice, whereas control mice were transfected with a scrambled plasmid (Fig. 6a, b, Supplementary Figure 7a). Two days after shRNA injection, we confirmed that *St8sia2* mRNA levels were specifically decreased in the amygdala, while *St8sia4* levels were not altered (Fig. 6c, Supplementary Figure 7b) and—as expected—not in the prefrontal cortex (Supplementary Figure 7c). Consistently, levels of polysialic acid were, in parallel, reduced in the amygdala (Fig. 6c) but not in the prefrontal cortex (Supplementary Figure 7c). As the *St8sia2* gene is developmentally downregulated from approximately P8-9 [29, 59] (Supplementary Figure 1a), our shRNA treatment from P2 efficiently decreases *St8sia2* expression until its endogenous developmental downregulation. Noteworthy, this treatment led as well to a significant decrease of GluN2B mRNA levels, without affecting GluN2A, as measured at P4 (Fig. 6d).

At adulthood, postnatal *St8sia2*-silencing in the amygdala led to pathological aggressive behaviors (Fig. 6e) while having no impact on body weight (Supplementary Figure 7d), anxiety traits (Supplementary Figure 7e), or social exploration (Supplementary Figure 7f). Furthermore, fear memory formation was impaired in *St8sia2*-silenced mice, which presented reduced freezing as compared to controls in the 24 h post-training cue test (Fig. 6f), while showing normal freezing levels to shock application during the training session (Fig. 6f).

This set of experiments demonstrates that postnatal *St8sia2* silencing in the amygdala is sufficient to cause aberrant aggression and impaired fear memory formation, along with a downregulation of GluN2B levels, and supports the link established in our previous experiments between these traits and *St8sia2-*induced amygdala dysfunction.

### PNS-induced alterations in St8sia2 and GluN2B expression levels

So far, our study implies that genetic modifications reducing *St8sia2* expression during early postnatal development lead to pathological aggression and can affect GluN2B expression in the amygdala leading to deficient synaptic plasticity. Next, we were interested in assessing whether prenatal environmental insults could affect expression for these genes. Indeed, evidence from the literature indicates that *St8sia2* expression can be modified by environmental experiences such as neonatal sensory deprivation or stress exposure [24–26]. Given that prenatal stress (PNS) can have strong programming behavioral effects [60], we measured gene expression in brains from embryos at embryonic day 18 (E18) derived from mothers submitted to unpredictable restraint stress from gestational day 14 (Fig. 6g). The *St8sia2* gene was downregulated in the stress group compared to non-stressed animals (Fig. 6h). On the contrary, there were no significant changes in *St8sia4* or NCAM genes. Strikingly, GluN2B levels were also downregulated in the stress group and correlated with *St8sia2* expression, which was not the case for GluN2A (Fig. 6i).

These data show that expression levels of *St8sia2* during early developmental periods—the time when the sialyltransferase coded by this gene is particularly prominent and highly relevant for proper brain development—are susceptible to be reduced by environmental insults, such as stress, that are known to exert important programming effects in brain and behavior.

## Discussion

Given the enormous damage and socioeconomic costs resulting from violence and the insufficiency of available treatments, understanding the mechanisms leading to abnormal aggression that could guide appropriate therapies is much needed. Here we show that *St8sia2*−/− mice are a valuable model for disentangling neurobiological mechanisms implicated in pathological aggression and for advancing on the rationale and validation of new treatments. In addition to recapitulating features of abnormal aggression, *St8sia2*−/− mice present key behavioral traits—impaired fear conditioning and reduced anxiety-like behaviors—strongly related to the occurrence of pathological aggression in humans [11, 12]. Deficits in fear learning are typically found in individuals with psychopathy [42, 61, 62] and hypothesized to promote antisocial behaviors, as they preclude individuals from learning from punishment and from following a normative socialization [63]. Deficits in fear processing also predict aggression in people with schizophrenia [13, 14] who, overall, show elevated risk for aggressive behavior and violent crime [6, 64]. Importantly, reminiscent of findings in humans with psychopathy, we underscore significant functional and structural alterations in the amygdala of *St8sia2*−/− mice. We also identify the amygdala as a critical brain region in which specific *St8sia2* deficiency leads to abnormal aggression and impaired fear learning. Furthermore, our data reveals a deficit in the GluN2B-containing NMDAR subunit underlying impaired fear memory-related long-term plasticity in the LA of *St8sia2*−/− mice. Based on these findings, we validate the partial NMDAR agonist DCS as an efficient drug to overcome long-term plasticity deficits in the LA and propose DCS treatment to reverse the behavioral aberrations associated with pathological aggression in these mice.

In line with their impairment in emotional processing, *St8sia2*−/− mice display functional—reduced pERK activation after a resident-intruder encounter and following fear conditioning—and structural—more thin, immature, and less mushroom-type, mature spines, as well as smaller excitatory synapses—alterations in the LA/BA. These findings are consistent with human imaging data indicative of amygdala atrophy [40] and blunted amygdala activation in association with deficits in fear processing in criminal psychopaths [41, 65–67] and in schizophrenia patients with comorbid psychopathy [48]. The reduction on threat-induced pERK activation in the LA/BA found in *St8sia2*−/− mice aligns with their reduced expression of glutamatergic genes (coding for GluN2A, GluN2B, and the synaptic marker Neuroligin1) that we find in these nuclei. At the protein level, lower GluN2B expression in these mice is found particularly circumscribed to the LA, where we consistently find reduced NMDAR currents with decreased GluN2B subunit content and impaired LTP. As opposed to the hippocampus, where GluN2B-containing receptors are highly expressed at extrasynaptic sites, in the LA they accumulate at the synapse and govern synaptic function and plasticity [68]. Our data are consistent with the implication of NMDARs [69]—particularly GluN2B-containing—on aggression-induced plasticity [70], fear conditioning [68, 71] and fear-induced pERK LA/BA activation [72]. By contrast, our genetic, structural and electrophysiological analyses indicate no alterations in the GABAergic transmission in LA. This suggests that amygdalar interneurons are less susceptible to polysialic acid-NCAM deficiency during early development as compared with cortical interneurons [73, 74].

We identify the amygdala as a critical region in which specific neonatal downregulation of *St8sia2* leads to abnormal aggression and impaired fear conditioning. St8sia2 expression levels in the brain are prominent during prenatal (from embryonic day 8) and early postnatal periods (Supplementary Figure 1) when its product, polysialic acid, is critically engaged in various neurodevelopmental processes, including neuronal proliferation and migration during embryogenesis and axonal growth, and synaptogenesis during postnatal period [27, 28]. Accordingly, *St8sia2*−/− mice show reduced—although not abolished, due to the remaining activity of the polysialyltransferase St8sia4—polysialic acid levels until around P9-P12[59]; from this period onwards, St8sia4 is the main polysialyltransferase controlling polysialic acid production in the brain. In the rodent BLA, the early neonatal period (in which our downregulation of *St8sia2* leads to aberrant behaviors) is characterized by a rapid synaptogenesis [75, 76] (and developmental processes leading to increased neuropil complexity [77]. This occurs particularly in principal glutamatergic neurons, as parvalbumin interneurons -a large percentage of interneurons in this brain region- start appearing in the BLA around P14 [78]. Thalamic and cortical inputs in the BLA have been reported to emerge from P7 [79] and functional maturation of the amygdala, including maturation of ionic currents to slowly emerge during the first weeks of life [80]. Therefore, a neonatal *St8sia2* reduction in the amygdala could have interfere with the development of synaptogenesis, dendritic complexity and, eventually, specific aspects of circuit development. In addition, although to our knowledge there was no previous study detailing the impact of *St8sia2* deficiency on neonatal amygdala development, several studies have revealed alterations in other brain regions occurring after birth in *St8sia2*−/− mice. The reported alterations include a decrease in GABA neurons in the prefrontal cortex observed at P1, but not E16.5; [81] deficits in olfactory interneurons only seen at P5; [82] and a decrease in myelination observed from P15 onwards [83]. More generally, polysialylation is an essential process involved in the migration and clustering of neural and glial precursors [23, 81, 84], axonal growth and fasciculation [85], synapse formation and plasticity [86, 87], and myelination [83].

Genetic association studies have identified that genes coding for NCAM and ST8SIA2 represent susceptibility loci for schizophrenia, autism and bipolar disorder [16–18, 88, 89], with a case study reporting violent behavior associated with *ST8SIA2* deletion [20]. Noteworthy, although we apply a genetic strategy here, *St8sia2* expression in the brain can also be diminished by environmental factors, such as sensory deprivation or stress exposure [24, 26, 90]. Importantly, we show that PNS, an environmental experience that in mice can lead to increased aggression [91] and impaired fear memory [92], leads to reduced *St8sia2* expression in the embryos’ brain. Thus, our PNS protocol from E14 to E18 seems to have a similar effect as the silencing of *St8sia2* (both constitutive and when specifically applied to the amygdala from P2). Strikingly, *St8sia2* correlated with GluN2B levels also in these embryonic brains. These observations imply that our findings in genetically modified mice (i.e., *St8sia2*−/− and amygdala-specific *St8sia2*-silenced mice) can, in addition, illuminate dysfunctional processes involving environmentally induced (e.g., PNS or other insults) *St8sia2* alterations. Altogether, our data are consistent with the notion that decreased polysialic acid levels in the amygdala during early development lead to downregulation of GluN2B expression. This, in turn, might preclude threat-induced plasticity and, consequently, prevent “learning from punishment” and interfering with normal socialization [63].

Prenatal *St8sia2* deficiency can interfere with neuronal migration and early synaptogenesis, whereas postnatal effects might particularly impinge upon synaptogenesis and neuropil developmental processes. GluN2B and *St8sia2* are both enriched in immature neurons [93, 94]. During development, the *St8sia2* product polysialic acid is downregulated to allow for synaptogenesis [73]. Therefore, *St8sia2* deficiency could lead to early neuronal maturation. This possibility seems plausible, particularly given the reported role of polysialic acid in GABAergic maturation in the visual cortex [74], leading to an earlier switch between GluN2A and 2B subunits. Further studies are needed to understand the structural and functional implications of the developmental reduction in St8sia2 expression impinged by PNS.

In addition to this developmental mechanism, we cannot totally exclude that some of our observed effects depend on potential actions of St8sia2 absence at adulthood. Although when globally quantified polysialic acid expression in the amygdala of *St8sia2*−/− mice is indistinguishable from the WT situation [94], there seems to be a small population of immature neurons expressing St8sia2 in the adult amygdala in mice [94]. These cells are most likely immature neurons and have been described in different species, including primates and humans [95, 96]. There is evidence indicating that these cells may progressively incorporate to amygdaloid circuitry, increasing the number of mature neurons; interestingly, this increase does not occur in autism spectrum disorder [97].

So far, pharmacological attempts to treat pathological aggression have been rather unsuccessful possibly due to the limited number of evidence-based treatments [3, 64]. Noteworthy, targeting the amygdala has been proposed as desirable aim to deal with psychopathy-related traits that predispose for pathological aggression [2]. Based on the mechanistic pathway identified in the amygdala in our study, we show that treatment with the partial NMDAR agonist DCS, both when administered i.c.v. and directly into the LA, is effective to normalize aberrant behaviors in *St8sia2*−/− mice related to abnormal aggression, blunted fear conditioning, and reduced anxiety-like behaviors. We also found that DCS facilitates NMDAR currents, restores LTP induction and enhances pERK expression in the LA, providing evidence that it corrects the impaired activation of GluN2B-containing NMDARs in the amygdala. Previously, DCS was shown to restore impaired hippocampal LTP and fear learning in NCAM-deficient mice [87]. In addition to facilitating fear extinction [98, 99], DCS has also been shown to facilitate fear memory consolidation in animals [100] and humans [101]. Therefore, our data put forward DCS as a potentially relevant treatment for abnormal aggression phenotypes coursing with impaired fear learning and reduced anxiety, a conjunction of traits frequently observed in people with elevated risk for aggressive behavior and violent crime [11–14].

Altogether, our study reveals a mechanistic rationale and preclinical pharmacological evidence supporting the relevance of translating our findings for the use of DCS to treat abnormal aggression in humans. In addition, our findings open new avenues for the exploration of the effectiveness of treatments targeting deficient polysialic acid levels [e.g., see ref. [102]] to overcome developmentally detected alterations associated with pathological aggression and predisposing traits.

## Electronic supplementary material

Supplemental Information

Supplementary Movie 1
