# Supplementary material for: Amygdala GluN2B-NMDAR dysfunction is critical in abnormal aggression of neurodevelopmental origin induced by St8sia2 deficiency
Source: Mol Psychiatry. 2018 Aug 8;25(9):2144–61. doi: 10.1038/s41380-018-0132-3 (PMC7473847; doi:10.1038/s41380-018-0132-3)
Supplement: Supplementary file 1 — Supplemental Information [file 41380_2018_132_MOESM1_ESM.pdf]

Supplementary materials

Amygdala GluN2B-NMDAR dysfunction is critical in abnormal aggression of neurodevelopmental origin induced by St8sia2 deficiency

Authors: Alexandre Bacq[1], Simone Astori[1], Elias Gebara[1], Wei Tang[2], Bianca A. Silva[3], Jose Sanchez-Mut[3], Jocelyn Grosse[1], Isabelle Guillot de Suduiraut[1], Olivia Zanoletti[1], Catherine Maclachlan[4], Graham W. Knott[4], Johannes Gräff[3] and Carmen Sandi[1]*

Affiliations:

[1]Laboratory of Behavioral Genetics, Brain Mind Institute, School of Life Sciences, EPFL, Lausanne, Switzerland

[2]Laboratory of Synaptic Mechanisms, Brain Mind Institute, EPFL, Lausanne, Switzerland

[3]Laboratory of Neuroepigenetics, Brain Mind Institute, EPFL, Lausanne, Switzerland

[4]Interdisciplinary Centre for Electron Microscopy, EPFL, Lausanne, Switzerland

*correspondence: carmen.sandi@epfl.ch

EPFL, SV-BMI-LGC,
SV 2805, Station 19,
CH-1015 Lausanne, Switzerland,
Tel: +41 21 693 95 34, Fax: +41 21 693 96 36

Supplementary Materials

Genotyping. All mice were genotyped before and after behavioral testing by polymerase chain reaction (PCR). DNA extracted from ear punches or phalanges with Accustart II protocol and submitted to PCR using the Supermix enzyme of QuantaBio (MA, USA), with use of the following primers: Forward primers for WT and KO: 5′-GGATAGTTACTGGGGTCAAGG-3′, and 5′-GCAGGTCGAGGGACCTAATA-3′, respectively; Reverse primer for both: 5′-AGACAGGAGGGTTTCGGAAT-3'.

Behavioral analyses. Behavioral characterization was performed in five cohorts of animals, (see **Results** section for more details). For the pERK experiments, cannulated and silenced animals, the behavioral schemes are detailed in **Results** section. Key behavioral experiments (resident-intruder and cue fear conditioning) were performed twice, and all attempts of replication were successful.

Auditory capacity. Startle response to acoustic stimuli was measured by a movement sensor with the SR-LAB^{TM} system (San Diego SA). After 3 days of habituation to the recording chamber, consisting of a tubular enclosure, with a background noise of 65 dB, animals were exposed to tones of different intensity, from 70 dB to 120 dB, for a duration of 40 ms.

Sensitivity to foot shock. This test was performed according to a previously published method.[1] Mice were individually placed in the conditioning chamber to receive 1-s shocks of gradually increasing current intensity by an increment of 0.01 mA, which typically elicited progressively increasing behavioral responses (flinching, 0.05–0.1 mA; jumping, 0.1–0.3 mA). The interval

between shocks was 20 s. The minimum current intensities required to elicit flinching and jumping in mice were measured.

Elevated plus maze. The maze consisted of two opposite open arms and two opposite closed arms (30 × 5 × 14 cm) arranged at right angles, and with a common central platform (5 × 5 cm) that gave access to all arms. Lighting was maintained at 15-16 lux on the open arms and 5-7 lux in the closed. The mouse was gently placed in a closed arm facing the wall and allowed to move undisturbed for 5 min. After each trial, the arms were cleaned with 10% ethanol and dried. A digital camera was mounted above the maze. Images were captured at a rate of 5 Hz and transmitted to a PC running the Ethovision (v. 3.1; Noldus Technology, Wageningen, The Netherlands) tracking system. The percentage of time spent in the open arms was taken as an indicator of anxiety. The total distance traveled in the entire surface (arms and central platform) of the maze provided a measure of general locomotor activity.

Open Field. Animals were placed in a rectangular arena (50 x 50 cm) and left to freely explore for 20 min. The light was adjusted to a level of 8-10 lux in the center of the arena. Video tracking of the animal's location was performed by a camera fixed above the arena, and images were transmitted at 5 Hz to a PC running Ethovision tracking system for further processing. The percentage of time spent in the center was taken as an indicator of anxiety.

Marble-burying test. To test the spontaneous burying behavior as an indication of anxiety-like behavior, mice were placed individually in a cage measuring 35 × 17 × 12 cm (L x W x H), containing bedding of 5 cm depth, with 12 glass marbles (2-3 cm diameter) evenly spaced on the

surface of the bedding. Testing was conducted for 20 min, and buried marbles (i.e. at least one-half covered with bedding) were counted at 1 min bins.

Locomotor activity. Animals were placed in individual new cages for a test duration of 24 hours. After 2 hours of habituation, locomotor activity was assessed by the number of laser breaks in PhenoMaster system (TSE Systems GmbH, Germany).

Social preference test. The sociability test was carried out in a three-chambered box (the center compartment was 20 × 35 × 35 cm and the left and right compartments were 30 × 35 × 35 cm). The dividing walls had retractable doorways that allowed access to each chamber. The test mouse was habituated to explore the entire apparatus for 10 min during 2 days. Each of the two side chambers contained an empty wire cage. The wire cages were 10 cm in height, with a bottom diameter of 9 cm and each bar spaced 1 mm apart. Juvenile mice (23 day-old C57BL/6J male mice) were habituated for 10 min to the wire cage for 2 days. In the third day (after habituation sessions), a test mouse was placed in the center compartment and allowed to explore the entire apparatus for 10 min. A juvenile mouse was enclosed in one of the wire cages, which was placed in one of the two sides of the social test box during the 10-min session. A dummy black mouse was placed in the other wire cage on the other side of the box. The time spent sniffing each wire cage was video-recorded and manually scored to evaluate the level of preference for the unfamiliar mouse compared with the object.

Immunohistochemistry

GABAergic and glutamatergic immunostaining. Sections were labeled with a goat anti-GAD67 (abcam ab80589, 1/500) and Rabbit anti-CamkII (abcam ab52476, 1/1000) to stain GABAergic

and glutamatergic neurons, respectively. Secondary antibodies were anti-Rabbit-alexa-488 and anti-goat-alexa-568 (abcam ab150073 and ab175474 respectively, 1/1000).

Quantification. Images were taken with confocal microscope (Zeiss LSM-700) using a 20X objective. Sample images were captured from different areas at the same coordinates for each animal using the mouse stereotaxic atlas as a reference.[2] Quantification was performed on original, unenhanced images only. Quantification of immunofluorescence LSM images were stitched together using the grid stitching plug-in for FIJI. The background intensity of each channel was measured at five different random areas and averaged to generate a mean background that was subtracted from each channel. Cells were delineated using a Triangle threshold to label only those stained with NeuN within 200-1000 pixels. The number of labeled cells that were co-labeled with phospho-ERK and the antibody of interest was counted and converted to a percentage of the total number of NeuN-stained cells for each section. Analyses were made blind to experimental conditions. GluN2A or GluN2B fluorescence intensities were measured in NeuN-positive cells.

Electrophysiological recordings.

The experiments described here were performed in 5-8 week-old mice.

Current-clamp recordings were performed with pipettes (2-3 MΩ) filled with an intracellular solution containing (in mM): 130 KGluconate, 10 KCl, 10 HEPES, 10 phosphocreatine, 0.2 EGTA, 4 Mg-ATP, 0.2 Na-GTP, (290-300 mOsm, pH 7.2-7.3). Resting membrane potential (V_{rmp}) was measured with no current injection, within 1 min from the establishment of the whole-cell configuration. Neuronal firing was induced by 2-s long depolarizing current steps (25 pA increments) from a membrane potential of -60 mV.

Miniature inhibitory postsynaptic currents (mIPSCs) were recorded in the presence of tetrodotoxin (1 μM), DNQX (10 μM) and D,L-APV (100 μM). Pipettes (2-3 MΩ) were filled with (in mM): 120 CsCl, 10 HEPES, 8 NaCl, 10 phosphocreatine, 0.2 $MgCl_2$, 0.2 EGTA, 2 Mg-ATP, 0.2 Na-GTP (290-300 mOsm, pH 7.2-7.3). Recordings were conducted at room temperature. Spontaneous events were acquired for 5 min at -60 mV, starting from >5 min after the establishment of the whole-cell configuration, to allow the diffusion of the intracellular solution. The contribution of perisomatic inhibition was evaluated by selecting the events with fast rise time (< 3 ms; this limit was chosen based on the cumulative distributions of all events, that display a relative peak below this value).[3,4]

To elicit asynchronous EPSCs (aEPSCs) at cortical inputs, extracellular $CaCl_2$ was replaced by equimolar $SrCl_2$. Cells were patched with the CsGluconate-based solution and held at -80 mV to amplify AMPAR-mediated currents. Only aEPSCs occurring between 20-500 ms after the onset of the first evoked EPSC were considered.

For detection of both mIPSCs and aEPSCs, traces were filtered at 1 kHz and analysed using the MiniAnalysis Program with a threshold corresponding to 2 times the baseline noise (Synaptosoft Inc., Decatur, USA).

Statistics. The number of animals/recordings per group was in agreement with the resource equation method to determine the sample size[5] and was guided by previous work of the lab with the same animal model. Power analysis was not performed, as animals typically underwent different experimental series (see **Supplementary Figure 2**), yielding multiple parameters with a group difference that was not known a priori. All groups consisted of males and were always matched per age among compared groups. For data exclusion, Grubbs' test for outliers was performed in GraphPad with an alpha level of 0.05, resulting in the exclusion of 1 animal from

the cue fear learning series, 1 animal from mRNA analysis, and of 1 datapoint in the LTP control series in WT animals. Electrophysiological data are presented with n numbers representing recordings pooled from at least 3 animals per series, to take into account inter-animal variability. Data from key experiments (such as AMPA/NMDA ratio, input-output curves, and LTP) were obtained from at least 5 animals. The animals used in this study were in general randomly distributed. Before any experiment, a number was allocated to each animal, and after all behavioral characterization and analyses, the genotype was uncovered. For the experiment involving treatment, trait anxiety of animals was measured as described in the methods, and subjects were assigned to control and drug groups in order to obtain groups with comparable mean anxiety. Data analysis was performed either by a researcher that was blind to genotype and treatment group or by an automated software.

Sample sizes are indicated in each Figure. The choice of parametric or nonparametric tests was based on normal distribution of the data (Shapiro-Wilk normality test). Unpaired two-tailed t test or Mann-Whitney test were used to compare sets of data obtained from independent groups of animals (WT vs. KO; Ct vs. sh; Ct vs stress). For two factors comparison, two-way analysis of variance (ANOVA) was used to analyze the effect of genotype, as well as treatment and interaction when applicable. Additional within-subject factors (e.g. CS-US, CS) were also included as determined by the nature of the dependent variables under consideration. Supplementary restricted analyses were also conducted to assist data interpretation whenever appropriate. Bonferroni and Fisher's LSD *post hoc* tests were used. All statistical analyses were performed with Prism 7 (Graphpad software Inc., San Diego, CA), expect for MANOVA which were performed with SPSS 11 (IBM, San Francisco). Electrophysiological data were analysed with Clampfit 10 (Molecular Devices), Igor 6 (Wavemetrics) and MiniAnalysis (Synaptosoft Inc.). Detailed parameters from statistical tests are reported in Figure legends or in the

Supplementary Table 2, with the second decimal rounded to the nearest value. Statistical significance was set at $\alpha < 0.05$.

References

1. Bourtchuladze R, Frenguelli B, Blendy J, Cioffi D, Schutz G, Silva AJ. Deficient long-term memory in mice with a targeted mutation of the cAMP-responsive element-binding protein. *Cell* 1994; **79**(1)**:** 59-68.

2. Franklin KB, Paxinos A. The mouse brain in stereotaxic coordinates. 1997.

3. Barsy B, Szabo GG, Andrasi T, Vikor A, Hajos N. Different output properties of perisomatic region-targeting interneurons in the basal amygdala. *Eur J Neurosci* 2017; **45**(4)**:** 548-558.

4. Veres JM, Nagy GA, Hajos N. Perisomatic GABAergic synapses of basket cells effectively control principal neuron activity in amygdala networks. *Elife* 2017; **6**.

5. Charan J, Kantharia ND. How to calculate sample size in animal studies? *J Pharmacol Pharmacother* 2013; **4**(4)**:** 303-306.

Supplemental Figures

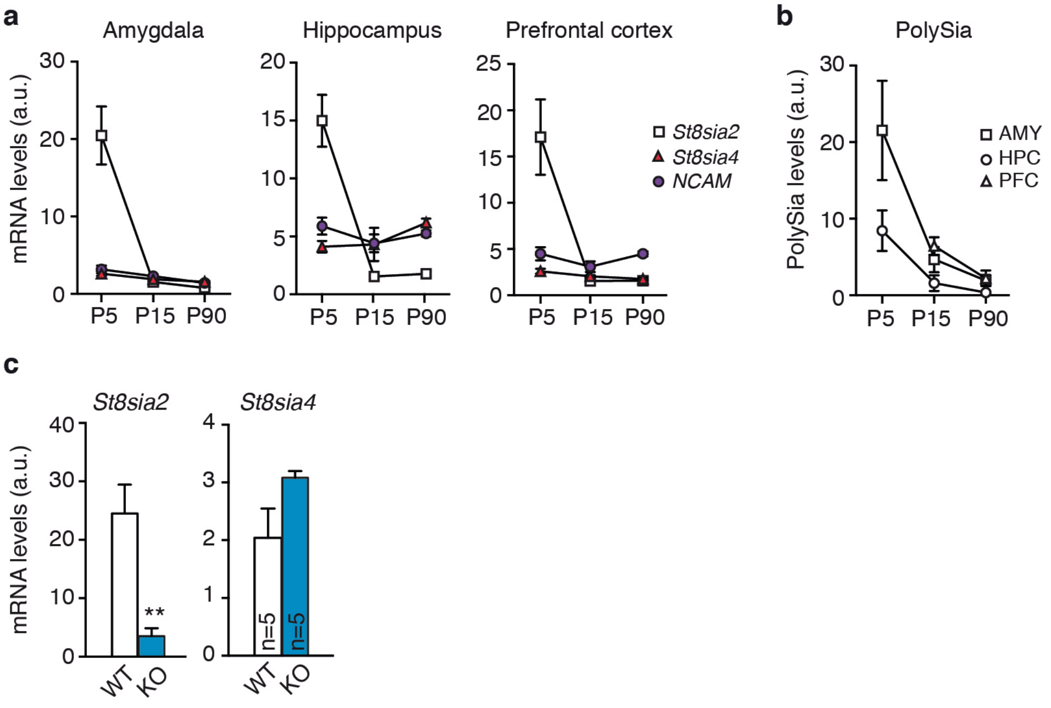

Supplementary Figure 1. Postnatal expression of Polysialic acid and its regulators in wild-type and *St8sia2-/-* mice. (**a**) Quantification of mRNA levels of *St8sia2*, *St8sia4* and *NCAM* at postnatal day 5 (P5), P15 and adulthood (P90) in wild-type mouse brain regions (n = 7 for P5, n = 6 for P15 and n = 7 for P90). (**b**) Quantification of Polysialic acid (PolySia) at different time points of development and adulthood in amygdala (AMY), hippocampus (HPC) and prefrontal cortex (PFC) (n=6 for P5, 7 for P15 and 8 for P90). (**c**) Quantification of mRNA levels of *St8sia2* (left) and *St8sia4* (right) in the amygdala of P4 *St8sia2-/-* (KO) mice compared to control (WT) littermates (unpaired t test, t_8=4.095, p=0.0035 for *St8sia2*; t_8=2.001, p=0.081 for *St8sia4*). **p<0.01.

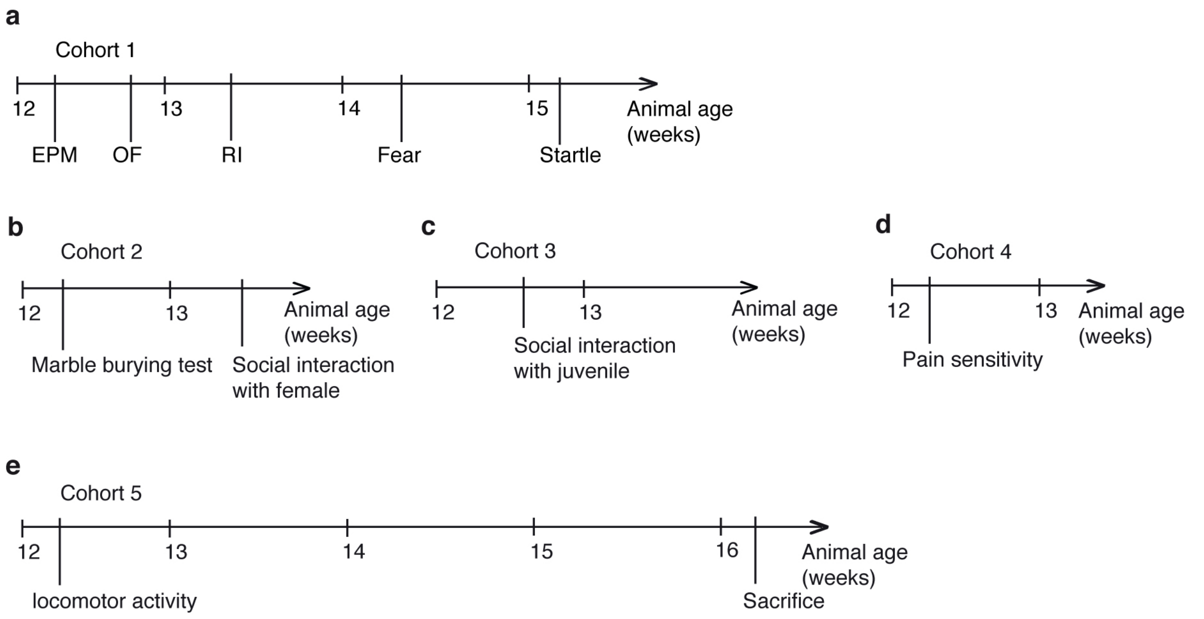

Supplementary Figure 2. Timelines of different experiments aiming at a behavioral characterization of *St8sia2-/-* mice. (**a**) Cohort 1 underwent tests for anxiety-related behavior (EPM: Elevated Plus Maze, OF: Open Field), resident-intruder (RI), Fear conditioning and Startle response. (**b**) Cohort 2 was tested for anxiety and social interaction with female. (**c-d**) Cohort 3 & 4 were tested for social interaction with juvenile and for pain sensitivity to foot shock, respectively. (**e**) Cohort 5 was tested for locomotor activity and gene expression.

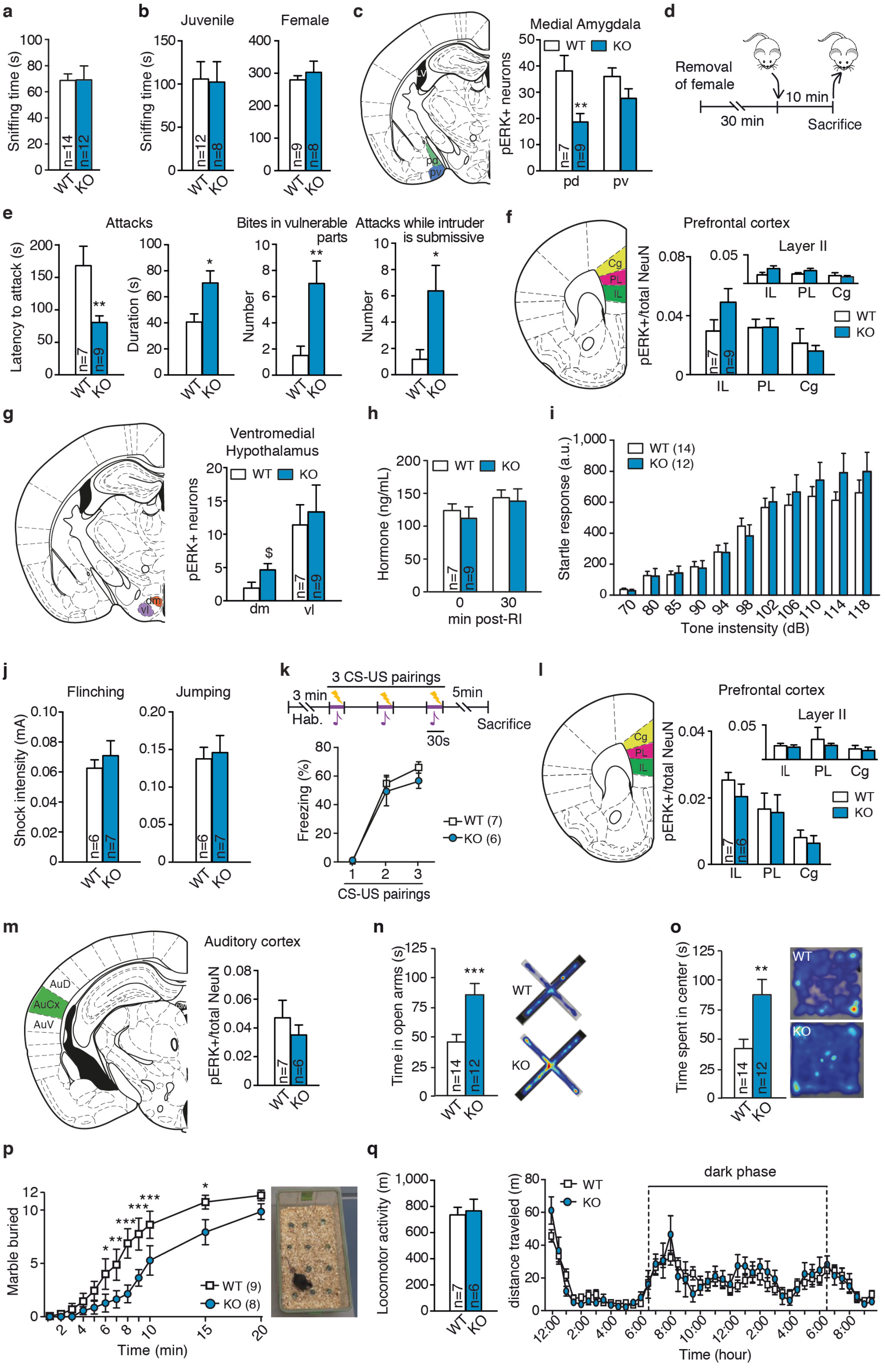
a
Sniffing time (s)
WT
KO
n=14
n=12
b
Juvenile
Female
Sniffing time (s)
n=12
n=8
n=9
n=8
c
Medial Amygdala
WT
KO
pERK+ neurons
n=7
n=9
pd
pv
d
Removal of female
30 min
10 min
Sacrifice
e
Attacks
Latency to attack (s)
Duration (s)
Bites in vulnerable parts
Attacks while intruder is submissive
Number
n=7
n=9
f
Prefrontal cortex
Layer II
Cg
PL
IL
pERK+/total NeuN
g
Ventromedial Hypothalamus
pERK+ neurons
dm
vl
h
Hormone (ng/mL)
min post-RI
i
Startle response (a.u.)
WT (14)
KO (12)
Tone instensity (dB)
j
Flinching
Jumping
Shock intensity (mA)
n=6
n=7
k
3 CS-US pairings
3 min
Hab.
5min
Sacrifice
30s
Freezing (%)
WT (7)
KO (6)
CS-US pairings
l
Prefrontal cortex
Layer II
pERK+/total NeuN
m
Auditory cortex
AuD
AuCx
AuV
pERK+/total NeuN
n
Time in open arms (s)
n=14
n=12
o
Time spent in center (s)
p
Marble buried
WT (9)
KO (8)
Time (min)
q
Locomotor activity (m)
n=7
n=6
dark phase
distance traveled (m)
Time (hour)

Supplementary Figure 3. Behavioral and immunohistochemical characterization of *St8sia2*-/- mice that complements Figure 1. (**a**) Social investigation of the intruder in the resident-intruder test. (**b**) Social investigation of juvenile or female conspecifics. (**c**) Localization and quantification of pERK activation after resident-intruder test in medial amygdala (pd: posterodorsal, pv: posteroventral) (unpaired t test, t_{14}=3.151, p=0.0062 for pd; t_{14}=1.576, p=0.13 for pv). (**d-e**) Characterization of abnormal aggression phenotype in the cohort in which pERK was quantified (**d**), including (in **e**) latency to attack (unpaired t test: t_{14}=3.089, p=0.009), duration of attacks (t_{14}=2.478, p=0.03), bites in vulnerable body parts (Mann-Whitney test: U=4, p=0.007) and number of attacks while the intruder is displaying submissive postures (Mann-Whitney test: U=5, p=0.012). (**f**) Localization and quantification of pERK activation after resident-intruder test in prefrontal cortex (PFC, IL: infralimbic, PL: prelimbic, Cg: cingulate). (**g**) Localization and quantification of pERK activation after resident-intruder test in ventromedial hypothalamus (VMH; dm: dorsomedial nucleus; vl: ventrolateral nucleus) (unpaired t-test: t_{14}=2.034, p=0.069 for dm; t_{14}=0.359, p=0.72 for vl). (**h**) Corticosterone levels measured just after resident-intruder, and 30 min later. (**i**) Auditory capacity assessed by startle responses to acoustic stimuli. (**j**) Pain sensitivity upon foot shock measured by flinching (left) and jumping (right). (**k**) Acoustic fear conditioning protocol (top) and corresponding freezing levels (bottom; two-way ANOVA: main effect of CS-US, $F_{2,22}$=96.58, p<0.0001; no effect of genotype and no interaction) used to investigate pERK activation. CS: conditioned stimulus (tone); US: unconditioned stimulus (foot shock); Hab.: habituation. (**l-m**) Assessment of pERK activation in the PFC (**l**) and in the auditory cortex (**m**) following training in the acoustic fear conditioning task. (**n-p**) Anxiety-related behaviors assessed in the elevated plus maze (**n**; unpaired t test, t_{24}=3.631, p=0.0006), open field (**o**; unpaired t test, t_{24}=3.11, p=0.0048) and marble burying test (**p**; two-way ANOVA: main effect of genotype, $F_{1,15}$= 8.25, p=0.0018, Bonferroni post hoc tests). (**q**) Locomotor activity measured in the home cage (two-way ANOVA, genotype factor: $F_{1,11}$=3.3, p=0.72). Results are given as mean ± s.e.m. $^{\$}$p<0.07; *p<0.05; **p<0.01; *** p<0.001 vs WT.

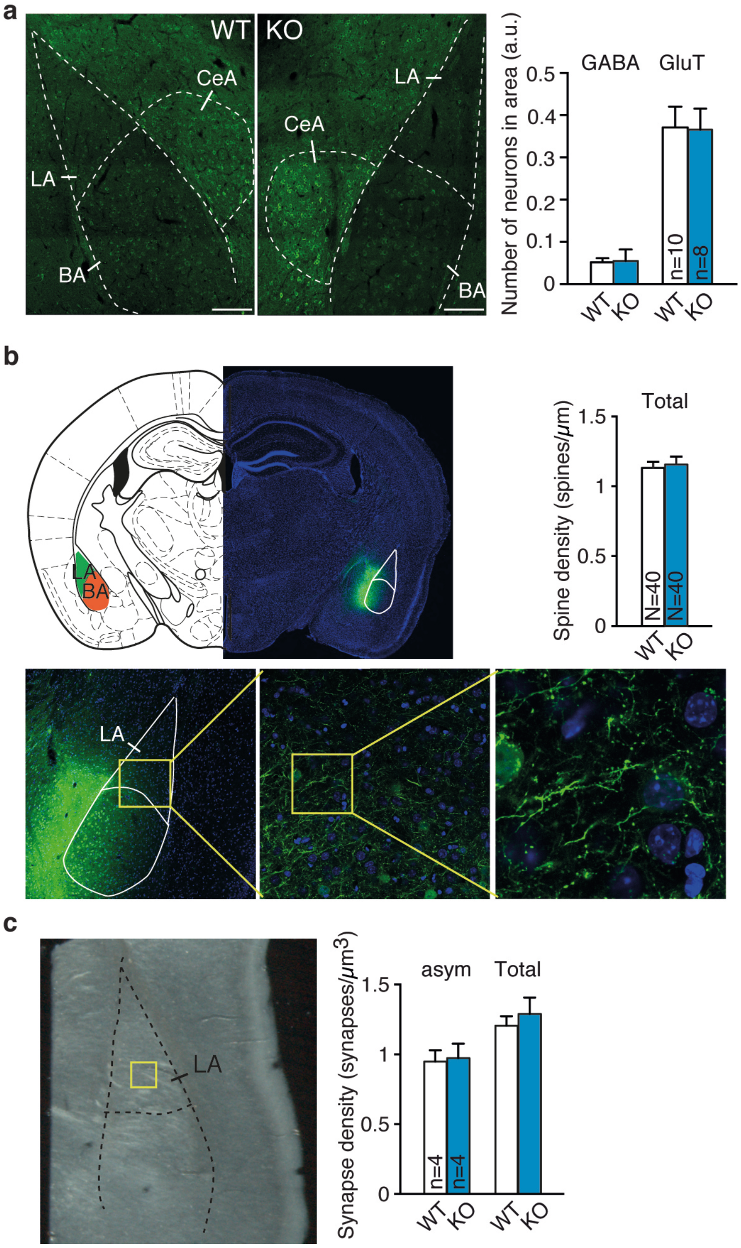

Supplementary Figure 4. Cellular composition and synaptic structure in the lateral amygdala that complement Figure 2. (**a**) Quantification of the GABAergic and glutamatergic neurons in the basolateral amygdala (right), with representative images (left) of GABAergic immunostaining in the amygdala of *St8sia2-/-* (KO) and wild-type (WT) mice (LA: lateral, BA: basal, CeA: central amygdala). (**b**) Morphological analyses of GFP-labeled neurons, with location of the injection site of GFP-lentivirus, with zoom in the lateral amygdala part analyzed. (**c**) Morphological analyses of electron microscopy, with location of the region analyzed (yellow rectangle). Results are given as mean ± s.e.m.

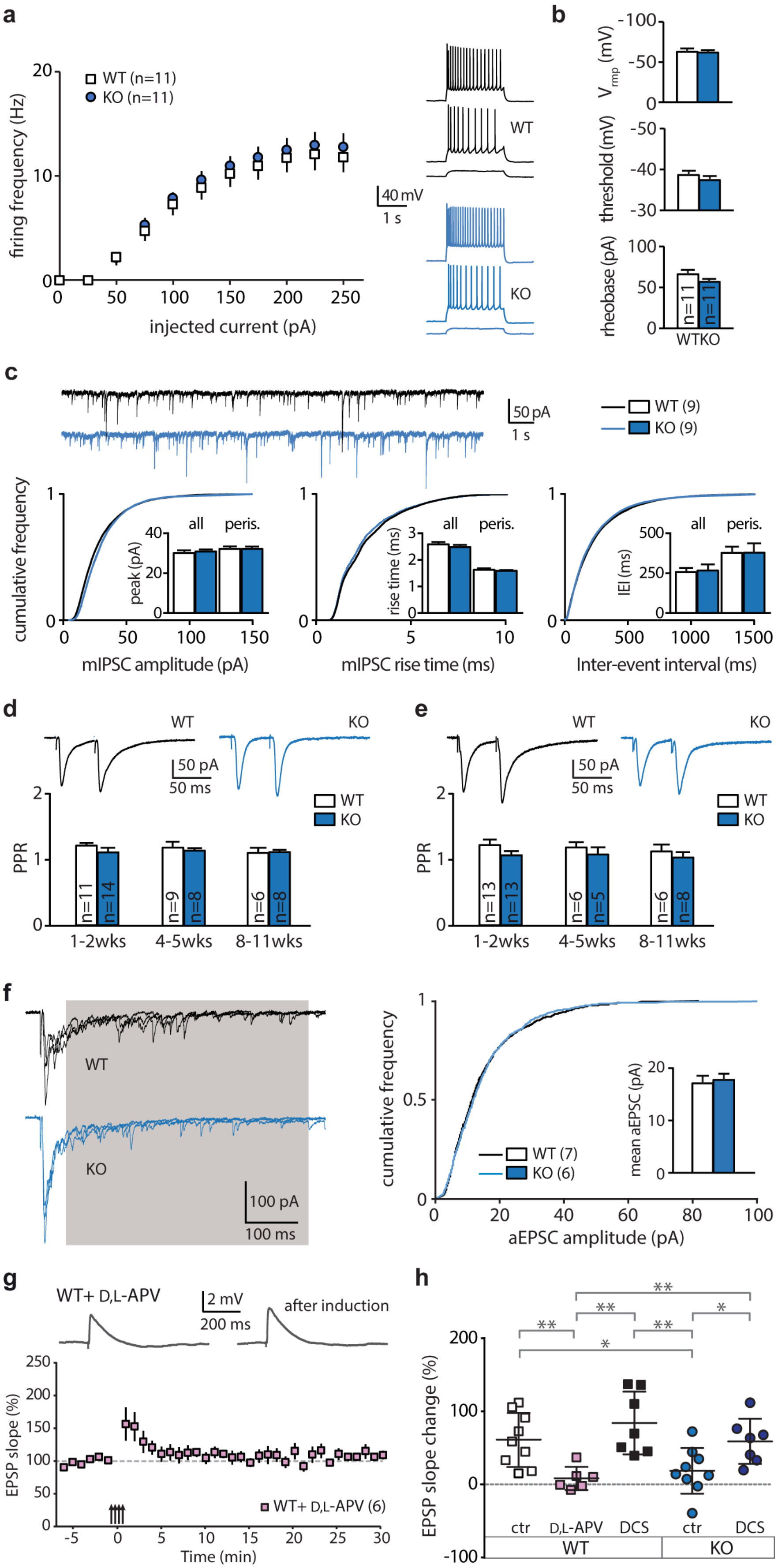

a
WT (n=11)
KO (n=11)
firing frequency (Hz)
injected current (pA)
WT
KO
40 mV
1 s
b
V_{rmp} (mV)
threshold (mV)
rheobase (pA)
n=11
n=11
WT
KO
c
50 pA
1 s
WT (9)
KO (9)
cumulative frequency
peak (pA)
all
peris.
rise time (ms)
IEI (ms)
mIPSC amplitude (pA)
mIPSC rise time (ms)
Inter-event interval (ms)
d
WT
KO
50 pA
50 ms
PPR
n=11
n=14
n=9
n=8
n=6
n=8
1-2wks
4-5wks
8-11wks
e
WT
KO
50 pA
50 ms
PPR
n=13
n=13
n=6
n=5
n=6
n=8
1-2wks
4-5wks
8-11wks
f
WT
KO
100 pA
100 ms
cumulative frequency
WT (7)
KO (6)
mean aEPSC (pA)
aEPSC amplitude (pA)
g
WT+ D,L-APV
2 mV
200 ms
after induction
EPSP slope (%)
WT+ D,L-APV (6)
Time (min)
h
EPSP slope change (%)
ctr
D,L-APV
DCS
ctr
DCS
WT
KO

Supplementary Figure 5. Electrophysiological analyses in *St8sia2*-/- and wild-type mice that complement Figure 3 and 4. (**a**) Frequency of firing elicited in LA pyramidal neurons by increasing somatic depolarizing currents did not differ between genotypes (two-way ANOVA, genotype: $F_{1,20}$=0.229, p=0.64). Color-coded traces on the right are representative voltage responses to steps of 25, 75 and 125 pA. (**b**) LA neurons from wild-type (WT) and *St8sia2*-/- (KO) mice did not differ for resting membrane potential (V_{rmp}; unpaired t test: t_{20}=0.2, p= 0.84), firing threshold (t_{20}=0.64, p=0.53), and rheobase (Mann-Whitney test, U=46.5, p=0.35). (**c**) Cumulative distributions of peak amplitude, rise time and inter-event interval of mIPSCs, showing no difference between genotypes (Kolmogorov-Smirnov test of cumulative distributions: peak, D=0.055, p=0.59; rise time, D=0.042, p=0.99; inter-event interval, D=0.017, p=0.34). Color-coded example traces are shown at the top. Bar graphs underneath the distributions represent mean values of the three parameters calculated from all events (all) and from events with fast rise time (<3 ms), reflecting mainly perisomatic inputs (peris.)(unpaired t test: p>0.05 for all parameters). (**d**-**e**) Paired-pulse ratio (PPR) of synaptic currents were measured throughout development at cortical (**d**) and subcortical (**e**) inputs (p>0.05 at all age windows). (**f**) Left, examples (superimposition of three single traces) of asynchronous release evoked at cortical inputs in the presence of Sr^{2+} (2 mM). Shaded area indicates the time window in which asynchronous excitatory postsynaptic currents (aEPSCs) were analysed. Right, cumulative distributions and mean peak amplitude (insets) of aEPSCs assessed in both genotypes (Kolmogorov-Smirnov test of cumulative distributions, D=0.029, p=0.73; Mann-Whitney test on mean peak values, U=14, p=0.36). (**g**) Hebbian LTP in WT mice was prevented by NMDAR blockade with 100 μM D,L-APV. (**h**) Summary of LTP recordings in WT and *St8sia2*-/- mice (One-way ANOVA: $F_{4,33}$=6.488, p=0.0006, followed by Fisher's LSD test). Results are given as mean ± s.e.m. *p<0.05; **p<0.01.

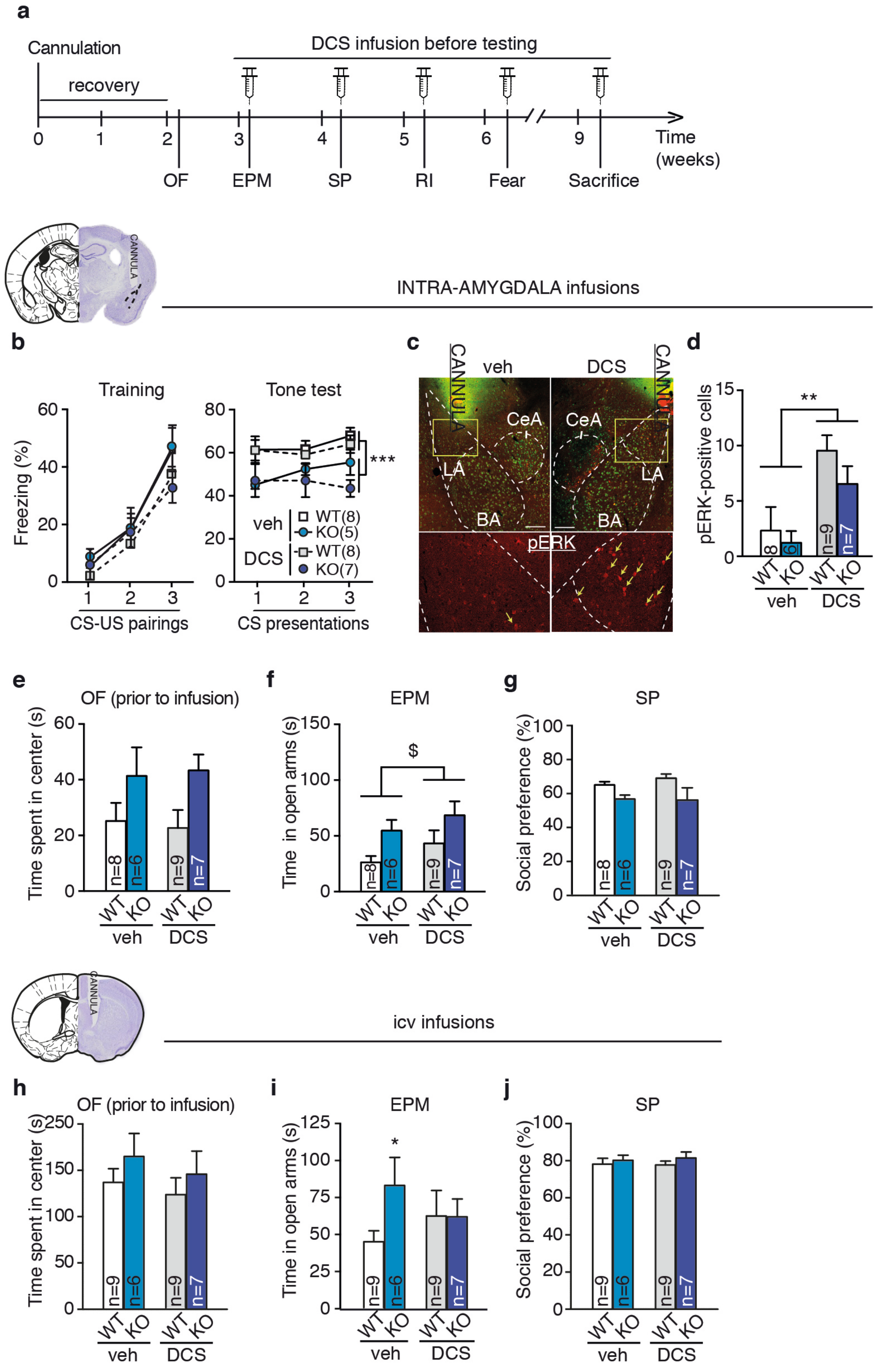

a
Cannulation
recovery
DCS infusion before testing
0 1 2 3 4 5 6 9
Time (weeks)
OF
EPM
SP
RI
Fear
Sacrifice
CANNULA
INTRA-AMYGDALA infusions
b
Training
Tone test
Freezing (%)
CS-US pairings
CS presentations
***
veh
WT(8)
KO(5)
DCS
WT(8)
KO(7)
c
veh
DCS
CANNULA
CeA
LA
BA
pERK
d
pERK-positive cells
**
n=8
n=6
n=9
n=7
WT KO WT KO
veh
DCS
e
OF (prior to infusion)
Time spent in center (s)
f
EPM
Time in open arms (s)
$
g
SP
Social preference (%)
icv infusions
h
OF (prior to infusion)
Time spent in center (s)
i
EPM
Time in open arms (s)
*
j
SP
Social preference (%)

Supplementary Figure 6. Behavioral effects of DCS injection intra-amygdala and in the lateral ventricle that complement Figure 5. (**a**) Protocol for both intracerebroventricular (icv) and intra-amygdala infusion of DCS (OF: Open-Field, EPM: Elevated Plus Maze, SP: Social preference, RI: resident-intruder). (**b**) Effect of intra-amygdala DCS infusion (20 min before acquisition of acoustic fear conditioning) on freezing behavior during training (left panel) and during the memory test (two-way ANOVA: main effect of genotype, $F_{1,24}$=13.75, p=0.001). (**c**) Representative images of pERK (red) and NeuN (green) colabeling in vehicle (veh) and DCS-infused animals (insets show pERK labeling of lateral amygdala, with activated neurons indicated by yellow arrows). Scale bars, 100 µm. (**d**) Quantification of pERK activation in LA (two-way ANOVA: effect of treatment: $F_{1,26}$=11.94, p=0.003). (**e-f, h-i**) Prior to DCS experiments, anxiety-related behavior was assessed after recovery from cannulation (**e, h**) in the OF test to balance groups assigned to vehicle (veh) and DCS with equivalent *a priori* levels of anxiety. (**e,** two-way ANOVA, main effect of genotype for intra-amygdala: $F_{1,26}$=5.42, p=0.029). After DCS or veh infusion, anxiety was tested in the elevated plus maze (EPM) (**f**, two-way ANOVA, main effect of genotype: $F_{1,26}$=7.84, p=0.011; effect for DCS: $F_{1,26}$=2.81, p=0.098; **i**, two-way ANOVA: interaction factor "treatment x genotype": $F_{1,27}$=3.51, p=0.05). (**g, j**) Social Preference (SP, **g**, for intra-amygdala infusion, two-way ANOVA reveals a main effect of genotype only: $F_{1,26}$=9.65, p=0.0056). Results are given as mean ± s.e.m. $^{\$}$p=0.098, *p<0.05, **p<0.01, ***p<0.001.

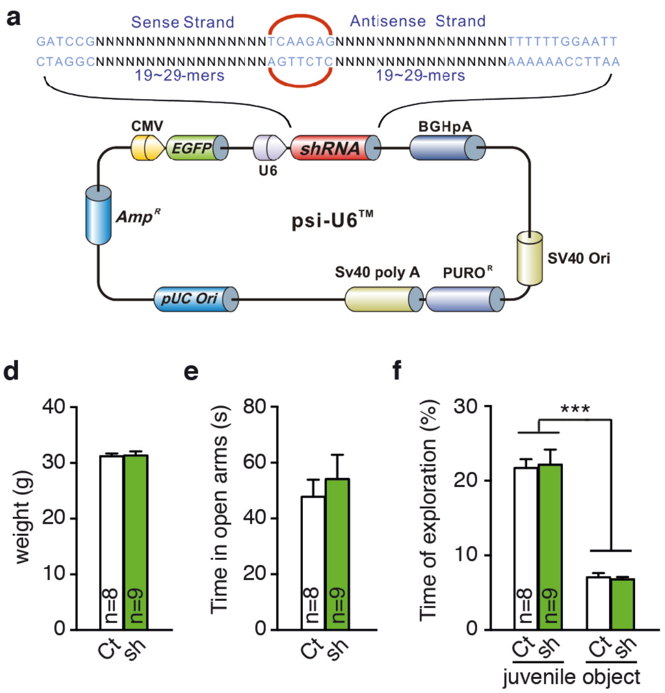

Supplementary Figure 7. Amygdala-restricted silencing of *St8sia2* during early development that complements Figure 6. (**a**) Design of the plasmid that was transfected *in vivo* through intra-amygdala infusion on postnatal day 2 (P2) in WT mice. (**b**) Quantification of mRNA levels of *St8sia4* in amygdala 2 days after injection of sh-St8sia2 plasmid (unpaired t-test: $t_8=0.024$, $p=0.98$). (**c**) Quantification of mRNA levels of *St8sia2* and Polysialic acid at P4 in the prefrontal cortex (PFC) of control (Ct) and silenced (sh) animals. (**d**) Body weight of adult animals (3 months old). (**e**) Anxiety-related behaviors assessed by the elevated plus maze. (**f**) Social preference test. Results are given as mean ± s.e.m. $^*p<0.05$; $^{***}p<0.001$.

Supplementary Table 1. Sequence of primers used for RT-qPCR. In green, the reference genes.

Name	RefSeq	Primer Forward	Primer Reverse
TBP	NM_013684.3	CTGGAATTGTACCGCAGCTT	CAGTTGTCCGTGGCTCTCTT
EEF1a1	NM_010106.2	TCCACTTGGTCGCTTTGCT	CTTCTTGTCCACAGCTTTGATGA
GAD67	NM_008077.4	CTTCTTCAGGCTCTCCCGTG	CAGGAACAGGCTCGGTTCAG
PV	NM_013645.3	TTTGCTGCTGCAGACTCCTT	AAGCCCTTCAGAATGGACCC
SST	NM_009215.1	GAAGATGCTGTCCTGCCGTC	AAGTACTTGGCCAGTTCCTGTT
vGAT	NM_009508.2	ACATTCATTATCAGCGCGGC	GCACGAACATGCCCTGAATG
GABAα1	NM_010250.5	ACACCATGAGGTTGACCGTG	TGCTACAACCACTGAACGGG
GABAα2	NM_008066.3	GGAAGCTACGCTTACACAACC	TGACTGGCCCAGCAAATCAT
GABAγ2	NM_008073.3	AGCCAGAAAATCTCTGCCA	GGGGCCTTGAAGGAAAACATC
Nlgn2	NM_198862.2	CCAAAGTGGGCTGTGACC	CCAAAGGCAATGTGGTAGC
vGlut1	NM_182993.2	TTGTGGCTACCTCCACCCTA	GCATAGGAACCGCAAAAGGC
vGlut2	NM_080853.3	GACTATGCGCAGAATCCGTC	CCAGCACCCTGTAGATCTGTC
GluN1	NM_008169.2	TGGTACCCATGTCATCCCAA	GCCATCACTCATTGTGGGCT
GluN2A	NM_008170.2	AAGAGCCTCATCACGCATGT	CGTGGATGTCGGATCCTTGT
GluN2B	NM_008171.3	TTCTGTCCCTTTATCCTCCGTCT	GCCAACACCAACCAGAACTT
GluN2C	NM_010350.2	TGTAATGTGCCTCACGGTGG	TGGTCCACCTGACTTCTTGC
GluN2D	NM_008172.2	GCTCAACTACATGGCCCGAA	CTCAATCTCATCGTCCCCCA
GluN3A	NM_001033351.2	ACGTGTGGAAAAGAGGTCCAA	TTGGTGGTCAGTGAAGCAGG
GluA1	NM_008165.4	GGGTCCGCCCTGAGAAATC	TCAGAGCACTGGTCTTGTCC
GluA2	NM_001083806.1	CAAGGACTCGGGAAGTAAGGAAA	CCAGCATTGCCAAACCAAGG
GluA3	NM_016886.4	CAGGCTCTCGAAAAGCTGGT	TGGTCCTGTTCTCGGAGGAT
mGluR1	NM_016976.3	AAACCCGAGAGGAATGTCCG	GCCGTTAGAATTGGCGTTCC
mGluR2	NM_001160353.1	GGTGCAGACCACTACGATGT	GGGATCCAGACCCTTGACCA
mGluR5	NM_001143834.1	AGCAACAATGAGCAGCTCCAA	ATGACTGCTGTCTGGTTGGG
mGluR7	NM_177328.3	AGAGAGCGACTGTGGAAGGA	CCAATTCGCTCCTGTCCTGT
Nlgn1	NM_001163387.1	GGGTACTTGGCTTCTTGAGCA	GGTTGACACATGAACCCCCA
St8sia2	NM_009181.2	TCGAAGAAGAAATCGGGAAT	GCGGTGAAGAGCCATTTATT
St8sia4	NM_009183.2	TCATCGGAGATGGTGAACTGTGT	ACAGAATGTTGGAAGATGGTGGAG
NCAM	NM_001081445.1	CACTGCCAGCAACACCAT	TGGTTCCCTTCCCAAGTGTA

Supplementary Table 2. Extensive statistical analyses.

Paradigm	Measurement	Statistical Test	Comparison	Statistics	p	Figure
Fear conditioning	Freezing in training	2-way ANOVA, with repeated measures for CS-US	Factor 1 genotype	$F_{1,24}=0.27$	0.60	1e
			interaction	$F_{2,48}=0.29$	0.74	
	Freezing in tone test	2-way ANOVA, with repeated measures for CS	Factor 2 CS	$F_{2,48}=0.61$	0.54	
			interaction	$F_{2,48}=1.35$	0.26	
pERK activation after Fear	CeA	unpaired t test	WT vs KO	$t_{11}=0.24$	0.81	1g
mRNA level	GAD67	unpaired t test	WT vs KO	$t_{11}=0.97$	0.35	2a
	PV	unpaired t test	WT vs KO	$t_{11}=1.54$	0.15	
	SST	unpaired t test	WT vs KO	$t_{11}=1.37$	0.19	
	vGAT	unpaired t test	WT vs KO	$t_{11}=0.72$	0.5	
	GABAα1	unpaired t test	WT vs KO	$t_{11}=0.12$	0.91	
	GABAα2	unpaired t test	WT vs KO	$t_{11}=0.76$	0.46	
	GABAγ2	unpaired t test	WT vs KO	$t_{11}=0.19$	0.84	
	Nlgn2	unpaired t test	WT vs KO	$t_{11}=0.23$	0.83	
	vGlut1	unpaired t test	WT vs KO	$t_{11}=1.07$	0.31	
	vGlut2	unpaired t test	WT vs KO	$t_{11}=1.179$	0.26	
	GluN1	unpaired t test	WT vs KO	$t_{11}=1.39$	0.19	
	GluN2C	unpaired t test	WT vs KO	$t_{11}=1.16$	0.27	
	GluN2D	unpaired t test	WT vs KO	$t_{11}=0.16$	0.88	
	GluN3A	unpaired t test	WT vs KO	$t_{11}=0.45$	0.42	
	GluA1	unpaired t test	WT vs KO	$t_{11}=0.17$	0.87	
	GluA2	unpaired t test	WT vs KO	$t_{11}=0.31$	0.76	
	GluA3	unpaired t test	WT vs KO	$t_{11}=0.03$	0.97	
	mGluR1	unpaired t test	WT vs KO	$t_{11}=0.68$	0.51	
	mGluR2	unpaired t test	WT vs KO	$t_{11}=1.34$	0.21	
	mGluR5	unpaired t test	WT vs KO	$t_{11}=1.4$	0.19	
	mGluR7	unpaired t test	WT vs KO	$t_{11}=0.08$	0.93	
Protein expression	GluN2A - LA	unpaired t test	WT vs KO	$t_{22}=0.81$	0.45	2b
	GluN2A - BA	unpaired t test	WT vs KO	$t_{22}=0.79$	0.46	
	GluN2A - CeA	unpaired t test	WT vs KO	$t_{22}=0.075$	0.94	
	GluN2B - BA	unpaired t test	WT vs KO	$t_{22}=1.67$	0.11	
	GluN2B - CeA	unpaired t test	WT vs KO	$t_{22}=2.11$	0.12	

AMPA/NMDA ratio at subcortical inputs	1-2 weeks	unpaired t test	WT vs KO	$t_{23}=0.427$	0.67	3d
	4-5 weeks	Mann-Whitney test	WT vs KO	U=18	0.7	
	8-11 weeks	Mann-Whitney test	WT vs KO	U=13	0.18	
	11-18 weeks	unpaired t test	WT vs KO	$t_{15}=0.39$	0.69	
Input-output curves	NMDA-currents at cortical inputs	2-way ANOVA	Factor 2 stimulation	$F_{7,98}=135.7$	<0.0001	3e
			interaction	$F_{7,98}=5.25$	<0.0001	
	AMPA-currents at cortical inputs	2-way ANOVA	Factor 1 genotype	$F_{1,14}=0.092$	0.76	
			Factor 2 stimulation	$F_{7,98}=65.8$	<0.0001	
			interaction	$F_{7,98}=0.064$	0.999	
	NMDA-current at subcortical inputs	2-way ANOVA	Factor 1 genotype	$F_{1,14}=0.096$	0.76	3f
			Factor 2 stimulation	$F_{6,84}=54.16$	<0.0001	
			interaction	$F_{6,84}=0.33$	0.917	
	AMPA-currents at subcortical inputs	2-way ANOVA	Factor 1 genotype	$F_{1,14}=0.386$	0.54	
			Factor 2 stimulation	$F_{6,84}=90.2$	<0.0001	
			interaction	$F_{6,84}=0.176$	0.982	
Resident-intruder	latency to attack	2-way ANOVA	Factor 1 genotype	$F_{1,24}=3.67$	0.067	5b
			Factor 2 treatment	$F_{1,24}=0.72$	0.41	
			interaction	$F_{1,24}=1.15$	0.29	
	Number of vulnerable bites	2-way ANOVA	Factor 1 genotype	$F_{1,24}=9.39$	0.0064	
			interaction	$F_{1,24}=2.84$	0.11	
	Number of attacks while intruder submissive	2-way ANOVA	Factor 1 genotype	$F_{1,24}=6.17$	0.023	
			interaction	$F_{1,24}=3.45$	0.079	
Fear conditioning	Freezing in training	MANOVA	CS	$F_{2,23}=43.5$	p<0.0001	5c
		MANOVA, Between Subjects effect	Factor 1 genotype	$F_{1,24}=1.31$	0.26	
			Factor 2 treatment	$F_{1,24}=0.56$	0.46	
			interaction	$F_{1,24}=1.85$	0.19	
	Freezing in tone test	MANOVA	CS	$F_{2,23}=0.31$	0.74	
		MANOVA, Between Subjects effect	Factor 1 genotype	$F_{1,24}=6.23$	0.02	
			Factor 2 treatment	$F_{1,24}=2.39$	0.14	
Resident-intruder	latency to attack	2-way ANOVA	Factor 1 genotype	$F_{1,26}=0.51$	0.48	5e
			Factor 2 treatment	$F_{1,26}=1.96$	0.18	
			interaction	$F_{1,26}=1.76$	0.2	
	Number of vulnerable bites	2-way ANOVA	Factor 1 genotype	$F_{1,26}=4.5$	0.051	
			Factor 2 treatment	$F_{1,26}=11.54$	0.004	

	Number of attacks while intruder submissive	2-way ANOVA	Factor 1 genotype	$F_{1,26}$=0.68	0.42	
			interaction	$F_{1,26}$=1.22	0.29	
Fear conditioning	Freezing in training	MANOVA	CS	$F_{2,19}$=23.62	p<0.0001	5f
		MANOVA, Between Subjects effect	Factor 1 genotype	$F_{1,20}$=0.22	0.64	
			Factor 2 treatment	$F_{1,20}$=0.096	0.76	
			interaction	$F_{1,20}$=0.123	0.73	
	Freezing in tone test	MANOVA	CS	$F_{2,19}$=2.79	0.093	
		MANOVA, Between Subjects effect	Factor 1 genotype	$F_{1,20}$=2.78	0.15	
			Factor 2 treatment	$F_{1,20}$=1.38	0.25	
Gene expression	*St8sia4*	unpaired t test	Ct vs stress	t_5=1.772	0.136	6d
Resident-intruder	latency - trial 1	unpaired t test	Ct vs sh	t_{15}=1.06	0.35	6e
	attacks while submissive	Mann-Whitney test	Ct vs sh	U=12	0.26	
Fear conditioning	Freezing in training	2-way ANOVA, with repeated measures for CS-US	Factor 1 genotype	$F_{1,15}$=0.34	0.56	6f
			interaction	$F_{2,30}$=0.61	0.55	
	Freezing in tone test	2-way ANOVA, with repeated measures for CS	Factor 2 tones	$F_{2,30}$=1.42	0.26	
			interaction	$F_{2,30}$=0.37	0.69	
Gene expression	*St8sia4*	unpaired t test	Ct vs stress	t_{11}=0.019	0.98	6h
	NCAM	unpaired t test	Ct vs stress	t_{11}=0.12	0.9	
	GluN2A	unpaired t test	Ct vs stress	t_{10}=0.71	0.5	6i

Supplementary Figures

RI	sniffing time	unpaired t test	WT vs KO	t_{24}=0.04	0.97	S3a
Social interaction	sniffing juvenile	unpaired t test	WT vs KO	t_{18}=0.1	0.91	S3b
	sniffing female	unpaired t test	WT vs KO	t_{15}=0.69	0.49	
pERK activation after RI	IL	unpaired t test	WT vs KO	t_{14}=1.42	0.18	S3f
	PL			t_{14}=0.036	0.97	
	Cg			t_{14}=0.61	0.55	
	IL - layer II			t_{14}=1.55	0.14	
	PL - Layer II			t_{14}=1.04	0.31	
	Cg- Layer II			t_{14}=0.48	0.64	
CORT after RI	+0 min	unpaired t test	WT vs KO	t_{14}=0.61	0.547	S3h
	+30 min	unpaired t test	WT vs KO	t_{14}=0.23	0.81	
Audition	Startle response	2-way ANOVA, with repeated measures for dB	Factor 1 genotype	$F_{1,24}$=0.49	0.48	S3i
			Factor 2 dB	$F_{10,240}$=48.83	p<0.0001	

			interaction	$F_{10,240}=0.94$	0.49	
Pain sensitivity	Flinching	unpaired t test	WT vs KO	$t_{11}=0.72$	0.48	S3j
	Jumping	unpaired t test	WT vs KO	$t_{11}=0.30$	0.76	
Fear conditioning	Freezing in training	2-way ANOVA, with repeated measures for CS-US	Factor 1 genotype	$F_{1,11}=0.71$	0.42	S3k
			interaction	$F_{2,22}=0.61$	0.56	
pERK activation after fear	CeA	unpaired t test	WT vs KO	$t_{11}=0.24$	0.81	S3l
	IL			$t_{11}=1.1$	0.31	
	PL			$t_{11}=0.14$	0.89	
	Cg			$t_{11}=0.51$	0.62	
	IL - layer II			$t_{11}=0.39$	0.71	
	PL - Layer II			$t_{11}=0.53$	0.61	
	Cg- Layer II			$t_{11}=0.36$	0.73	
pERK activation after Fear	AuCx	unpaired t test	WT vs KO	$t_{11}=0.85$	0.42	S3m
marble burying test	Marble buried	2-way ANOVA, with repeated measures for time	Factor 2 Time	$F_{11,165}=116$	p<0.0001	S3p
			interaction	$F_{11,165}=6.31$	p<0.0001	
Locomotor activity	Total distance traveled	unpaired t test	WT vs KO	$t_{11}=0.31$	0.76	S3q
Locomotor activity	distance traveled	2-way ANOVA, with repeated measures for Time	Factor 2 Time	$F_{43,473}=7.92$	p<0.0001	
			interaction	$F_{43,473}=0.84$	0.75	
Number of neurons	GABAergic	unpaired t test	WT vs KO	$t_{16}=0.12$	0.91	S4a
	Glutamatergic	unpaired t test	WT vs KO	$t_{16}=0.079$	0.94	
Morphological composition of LA, with GFP-neurons	Total synapse	unpaired t test	WT vs KO	$t_{2}= 2.819$	0.11	S4b
Morphological composition of LA, with EM	asymmetric	unpaired t test	WT vs KO	$t_{6}=0.19$	0.85	S4c
	total	unpaired t test	WT vs KO	$t_{6}=0.61$	0.56	
Firing	Firing	2-way ANOVA	Factor 2 current	$F_{10,200}=139.9$	p<0.0001	S5a
			interaction	$F_{10,200}=0.213$	0.995	
mIPSCs amplitude	All	unpaired t test	WT vs KO	$t_{16}=0.27$	0.78	S5c
	peris	unpaired t test	WT vs KO	$t_{16}=0.014$	0.99	
mIPSCs rise time	All	unpaired t test	WT vs KO	$t_{16}=0.64$	0.53	
	peris	unpaired t test	WT vs KO	$t_{16}=0.94$	0.36	
mIPSCs inter event interval	All	unpaired t test	WT vs KO	$t_{16}=0.18$	0.85	
	peris	unpaired t test	WT vs KO	$t_{16}=0.023$	0.98	
Paired Pulse ratio at cortical inputs	1-2 weeks	unpaired t test	WT vs KO	$t_{23}=1.26$	0.22	S5d
	4-5 weeks	Mann-Whitney test	WT vs KO	U=33.5	0.83	

	8-11 weeks	Mann-Whitney test	WT vs KO	U=22	0.83	
Paired Pulse ratio at subcortical inputs	1-2 weeks	unpaired t test	WT vs KO	$t_{24}=1.47$	0.15	S5e
	4-5 weeks	Mann-Whitney test	WT vs KO	U=10	0.39	
	8-11 weeks	Mann-Whitney test	WT vs KO	U=18.5	0.51	
Fear conditioning intra-AMY	Freezing in training	MANOVA	CS-US	$F_{2,23}=55.72$	$p<0.0001$	S6b
		MANOVA, Between Subjects effect	Factor 1 genotype	$F_{1,24}=0.039$	0.84	
			Factor 2 treatment	$F_{1,24}=2.97$	0.098	
			interaction	$F_{1,24}=0.033$	0.86	
	Freezing in tone test	MANOVA	CS	$F_{2,23}=0.79$	0.46	
		MANOVA, Between Subjects effect	Factor 2 treatment	$F_{1,24}=0.94$	0.34	
			interaction	$F_{1,24}=0.14$	0.71	
pERK activation	LA	2-way ANOVA	Factor 1 genotype	$F_{1,26}=1.76$	0.20	S6d
			interaction	$F_{1,26}=0.64$	0.44	
Open field intra-AMY	Time in center	2-way ANOVA	Factor 2 group	$F_{1,26}=0.0009$	0.98	S6e
			interaction	$F_{1,26}=0.079$	0.78	
elevated plus maze intra-AMY	time in open arms	2-way ANOVA	interaction	$F_{1,26}=0.0003$	0.99	S6f
social preference intra-AMY	social preference	2-way ANOVA	Factor 2 treatment	$F_{1,26}=0.24$	0.63	S6g
			interaction	$F_{1,26}=0.44$	0.51	
Open field icv	Time in center	2-way ANOVA	Factor 1 genotype	$F_{1,27}=1.59$	0.22	S6h
			Factor 2 group	$F_{1,27}=0.67$	0.42	
			interaction	$F_{1,27}=0.015$	0.90	
Elevated plus maze icv	Time of interaction	2-way ANOVA	Factor 1 genotype<br>Factor 2 treatment	$F_{1,27}=3.76$<br>$F_{1,27}=0.37$	0.063<br>0.55	S6i
social preference icv	social preference	2-way ANOVA	Factor 1 genotype	$F_{1,27}=0.94$	0.34	S6j
			Factor 2 treatment	$F_{1,27}=0.021$	0.88	
			interaction	$F_{1,27}=0.077$	0.78	
St8sia2 mRNA levels	PFC	unpaired t test	Ct vs sh	$t_{8}=0.28$	0.79	S7c
PolySia level	PFC	unpaired t test	Ct vs sh	$t_{8}=0.35$	0.73	
Body weight	distance moved	unpaired t test	Ct vs sh	$t_{15}=0.14$	0.88	S7d
elevated plus maze	time in open arms	unpaired t test	Ct vs sh	$t_{15}=0.73$	0.44	S7e
social preference	Time of interaction	2-way ANOVA	Factor 1 genotype	$F_{1,30}=0.0048$	0.95	S7f
			Factor 2 juvenile-object	$F_{1,30}=144.5$	$p<0.0001$	
			interaction	$F_{1,30}=0.09$	0.76	
